# Genetic Control of Conventional and Pheromone-Stimulated Biofilm Formation in *Candida albicans*

**DOI:** 10.1371/journal.ppat.1003305

**Published:** 2013-04-18

**Authors:** Ching-Hsuan Lin, Shail Kabrawala, Emily P. Fox, Clarissa J. Nobile, Alexander D. Johnson, Richard J. Bennett

**Affiliations:** 1 Department of Microbiology and Immunology, Brown University, Providence, Rhode Island, United States of America; 2 Department of Microbiology and Immunology, University of California, San Francisco, San Francisco, California, United States of America; 3 Tetrad Program, Department of Biochemistry and Biophysics, University of California, San Francisco, San Francisco, California, United States of America; University of Aberdeen, United Kingdom

## Abstract

*Candida albicans* can stochastically switch between two phenotypes, white and opaque. Opaque cells are the sexually competent form of *C. albicans* and therefore undergo efficient polarized growth and mating in the presence of pheromone. In contrast, white cells cannot mate, but are induced – under a specialized set of conditions – to form biofilms in response to pheromone. In this work, we compare the genetic regulation of such “pheromone-stimulated” biofilms with that of “conventional” *C. albicans* biofilms. In particular, we examined a network of six transcriptional regulators (Bcr1, Brg1, Efg1, Tec1, Ndt80, and Rob1) that mediate conventional biofilm formation for their potential roles in pheromone-stimulated biofilm formation. We show that four of the six transcription factors (Bcr1, Brg1, Rob1, and Tec1) promote formation of both conventional and pheromone-stimulated biofilms, indicating they play general roles in cell cohesion and biofilm development. In addition, we identify the master transcriptional regulator of pheromone-stimulated biofilms as *C. albicans* Cph1, ortholog of *Saccharomyces cerevisiae* Ste12. Cph1 regulates mating in *C. albicans* opaque cells, and here we show that Cph1 is also essential for pheromone-stimulated biofilm formation in white cells. In contrast, Cph1 is dispensable for the formation of conventional biofilms. The regulation of pheromone- stimulated biofilm formation was further investigated by transcriptional profiling and genetic analyses. These studies identified 196 genes that are induced by pheromone signaling during biofilm formation. One of these genes, *HGC1*, is shown to be required for both conventional and pheromone-stimulated biofilm formation. Taken together, these observations compare and contrast the regulation of conventional and pheromone-stimulated biofilm formation in *C. albicans*, and demonstrate that Cph1 is required for the latter, but not the former.

## Author Summary

*Candida albicans* is the predominant fungal pathogen afflicting humans, where many infections arise due to its proclivity to form biofilms. Biofilms are complex multicellular communities in which cells exhibit distinct properties to those grown in suspension. They are particularly relevant in the development of device-associated infections, and thus understanding biofilm regulation and biofilm architecture is a priority. *C. albicans* has the ability to form different types of biofilms under different environmental conditions. Here, we compare the regulation of biofilm formation in conventional biofilms, for which a core transcriptional network has recently been identified, with pheromone-stimulated biofilms, which occur when *C. albicans* white cells are exposed to pheromone. Our studies show that several regulatory components control biofilm formation under both conditions, including the network transcriptional regulators Bcr1, Brg1, Rob1, and Tec1. However, other transcriptional regulators are specific to each model of biofilm development. In particular, we demonstrate that Cph1, the master regulator of the pheromone response during mating, is essential for pheromone-stimulated biofilm formation but is dispensable for conventional biofilms. These studies provide an in-depth analysis of the regulation of pheromone-stimulated biofilms, and demonstrate that both shared and unique components operate in different models of biofilm formation in this human pathogen.

## Introduction

*Candida albicans* is a prevalent pathogen of humans that colonizes and infects multiple niches in the mammalian host. To achieve such extreme adaptability, this pathogen has evolved genetic and epigenetic mechanisms that modulate cell behavior and morphology in response to environmental signals. Epigenetic variation in *C. albicans* is perhaps best exemplified by the white-opaque phenotypic switch. This is a heritable and reversible switch in which cells transition between white cells that are round and give rise to dome-shaped, shiny colonies, and opaque cells that are elongated and give rise to flatter, darker colonies [1]. Switching is regulated by a core circuit of transcription factors that operate within a network of positive and negative feedback loops [2], [3]. Similar transcriptional networks are found in many biological systems and act to regulate developmental programs from yeast to mammals [3], [4].

White and opaque cells exhibit striking behavioral differences, including their contrasting ability to undergo sexual reproduction. Opaque cells are the mating competent form of *C. albicans* and secrete sex-specific pheromones that induce mating responses in cells of the opposite mating type [5]. Pheromone signaling in opaque cells leads to the upregulation of genes required for cell and nuclear fusion, as well as the formation of polarized mating projections [6]–[8]. In contrast, white cells are refractory to mating, undergoing **a**-α cell fusion at least a million times less efficiently than opaque cells [5]. However, white **a** or α cells become adherent in response to pheromones secreted by opaque cells, leading to enhanced biofilm formation [9]. It is speculated that such pheromone-stimulated biofilms could increase mating between opaque cells by stabilizing pheromone gradients and promoting chemotropism between rare mating partners [9]. Biofilms also represent a significant threat for the development of clinical infections by *C. albicans*. These surface-associated communities can form on implanted medical devices and host surfaces, and are resistant to antifungal treatment, while also promoting the seeding of serious bloodstream infections [10], [11].

“Conventional” biofilms are formed when *C. albicans* yeast cells adhere to a surface followed by maturation due to pseudohyphae and hyphae formation and production of extracellular matrix material [10], [12]. Hyphae formation is an important feature of biofilms as mutants blocked in filamentation are often impaired in biofilm development [12]. The core transcriptional network regulating conventional biofilms has recently been elucidated and, similar to the white-opaque switch, involves interacting transcriptional feedback loops [13]. Six transcription regulators were shown to operate the biofilm regulatory network including Bcr1, Brg1, Efg1, Rob1, Ndt80, and Tec1 [13]. Loss of any one of these regulators significantly compromised biofilm formation *in vitro*, and these factors were also necessary in two *in vivo* animal models of biofilm formation [13]. This work was carried out in **a**/α cells, and these biofilms were formed by exposing *C. albicans* to a solid surface (bovine serum-coated polystyrene or silicone substrates) and allowing the biofilm to form over the course of 24 to 48 hours, with gentle shaking of the samples at physiological temperature (37°C).

Recent studies have begun to address the regulation of biofilm formation in pheromone-stimulated (or sexual) biofilms, and to compare mechanisms of pheromone signaling in white and opaque cells. In this case, the biofilms are formed from white **a** or α cells without shaking or on a slow rocker at 25–29°C. Under these conditions, significantly thicker biofilms are formed by **a** cells in the presence of α pheromone, or by α cells in the presence of **a** pheromone [9], [14], [15]. Pheromone responses in diverse yeast species are mediated by a conserved G-protein coupled MAPK cascade that culminates in transcription factor activation [16]. Studies have established that the *C. albicans* transcription factor Cph1 (ortholog of *S. cerevisiae* Ste12) is activated by MAPK signaling and mediates expression of mating genes in opaque cells [17]–[19]. However, in contrast to this paradigm (which also holds true for *S. cerevisiae*, *Kluyveromyces lactis*, and *Candida lusitaniae* [20]–[22]), pheromone signaling in *C. albicans* white cells was proposed to activate a different transcription factor, Tec1, with Cph1 dispensable for signaling in this cell type [14].

In this manuscript, we compare and contrast the genetic requirements for conventional and pheromone-stimulated biofilms, and re-address the role of Cph1 in these processes. We show that four of the six transcriptional regulators of conventional biofilm formation (Bcr1, Brg1, Rob1, and Tec1) are also necessary for pheromone-stimulated biofilms. However, in contrast to previous reports, we demonstrate that Cph1 is the master transcription factor mediating MAPK signaling in white and opaque cells of *C. albicans*. Thus, Cph1 is essential for pheromone-stimulated biofilm formation in white cells as well as sexual mating in opaque cells. Transcriptional profiling of pheromone-stimulated biofilms was also performed and provides the first genome-wide picture of this developmental program. Gene expression profiles of wildtype, *Δcph1/Δcph1*, and *Δtec1/Δtec1* strains were compared, and confirmed that Cph1 is essential for the transcriptional response to pheromone. Downstream targets of Cph1 were identified including Hgc1, which is shown to play a significant role in both pheromone-stimulated and conventional biofilms. Overall, our data reveals that several components of biofilm regulation are shared between conventional and pheromone-stimulated biofilm models, but that other transcription factors operate specifically in only one program of biofilm development.

## Results

### Comparison of Conventional and Pheromone-Stimulated Biofilms

In order to compare the genetic requirements for different types of biofilms formed under different conditions, we performed an experiment where we directly compared two distinct biofilm models using isogenic strains. These experiments were carried out in two different laboratories and used multiple independent mutants to confirm the findings.

**Figure 1:**
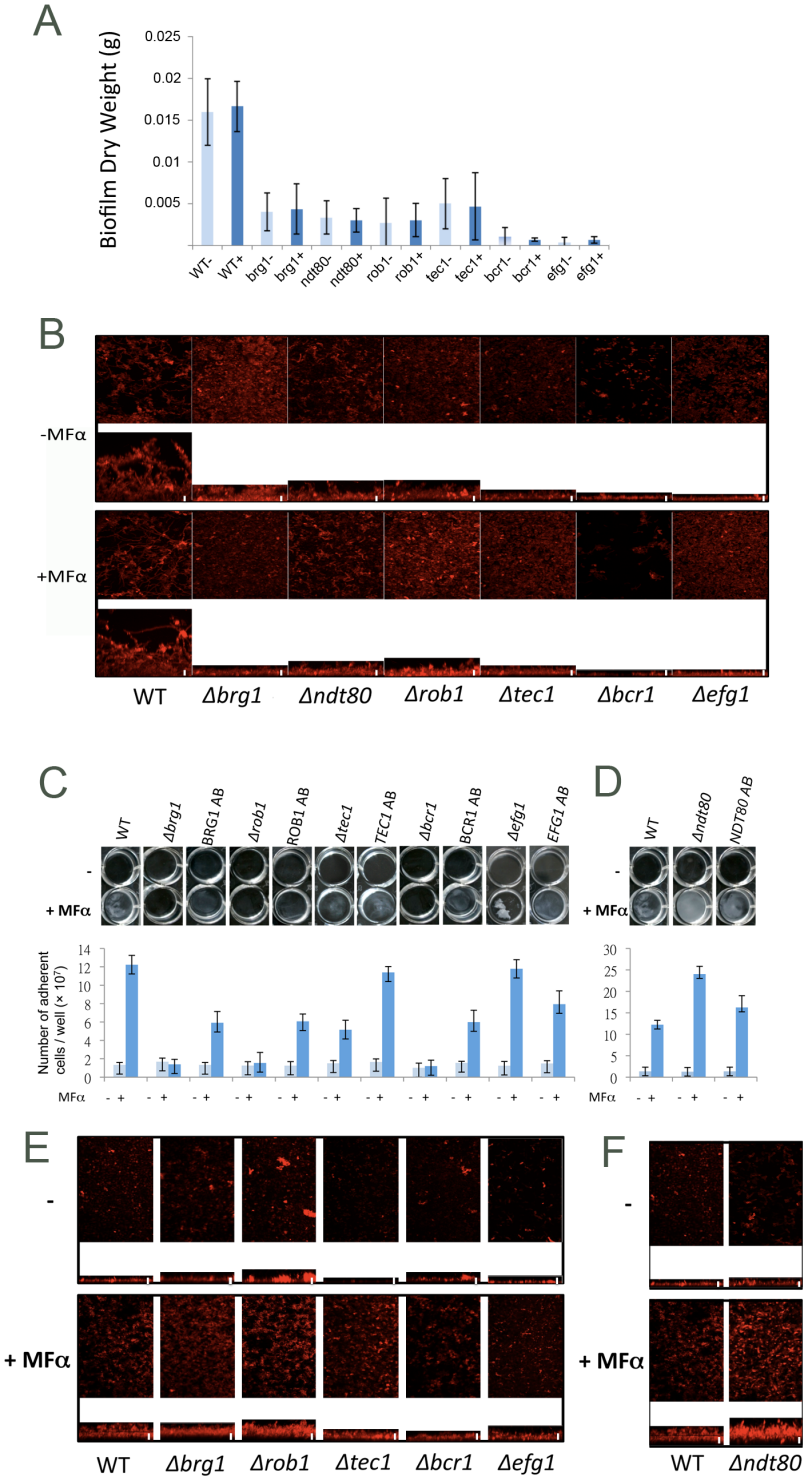
Comparison of the Genetic Regulation of Conventional and Pheromone-Stimulated Biofilms. Analysis of the role of six biofilm transcription factors, Bcr1, Brg1, Efg1, Ndt80, Rob1, and Tec1, in conventional and pheromone-stimulated biofilm formation, in the presence (+) and absence (−) of α pheromone (MFα). P37005 cells (or mutant derivatives) were grown as conventional biofilms in Spider medium with shaking at 37°C and samples analyzed by (A) biofilm dry weights, and (B) confocal scanning laser microscopy (CSLM). P37005 cells (or mutant derivatives) were grown as pheromone-stimulated biofilms in Lee's medium without shaking at 25°C and samples analyzed by the number of adherent cells (C, D) and by CLSM (E, F). All mutant strains were significantly reduced in conventional biofilm formation (*P*<0.05). Mutants in Brg1, Rob1, Tec1, and Bcr1 were also significantly reduced in pheromone-stimulated biofilm formation (*P*<0.01). Values are the mean ± SD from two independent experiments with at least three replicates. AB indicates addback of one copy of the deleted gene to the corresponding mutant. Light blue bars on graphs, no pheromone added. Dark blue bars on graphs, pheromone present. For each CSLM, the top panel represents the top view while the bottom panel represents the side view. Scale bars in CSLM images are 20 µm.

Figure 1A and 1B shows the results of a series of isogenic white **a** strains tested under the set of biofilm conditions described in Nobile *et al.* [13]. These biofilms were formed at 37°C in Spider medium with shaking. The results show, by cell number, dry weight and confocal scanning laser microscopy (CSLM), that deletion of any one of the six core transcription regulators (Bcr1, Brg1, Efg1, Tec1, Ndt80, or Rob1), severely reduced biofilm formation (Figure 1A and B, and data not shown). These results are consistent with those of Figure 1 in Nobile *et al.*, the only difference being that the experiments described here were performed with white *MTL* **a** cells while Nobile *et al.* used white *MTL* **a**/α cells. Addition of pheromone under these conditions did not produce any apparent differences either in the dry weights or in the appearance of the biofilm by CSLM (Figure 1A and B). These experiments were conducted in the *C. albicans* SC5314 strain background and we will refer to this protocol as the “conventional” biofilm assay.

Figure 1C–F shows the same set of strains subjected to a different type of biofilm assay, first described by Daniels *et al.* [9]. Here, biofilms were formed at room temperature in Lee's medium without shaking. Under these conditions, wildtype white **a** cells formed a very weak biofilm in the absence of pheromone, and α pheromone treatment significantly increased biofilm formation (Figure 1C and D). We will refer to this protocol as the “pheromone-stimulated” biofilm assay. We note that biofilms produced by SC5314-derived strains under these conditions are more fragile than those produced under the “conventional” biofilm assay and that they adhere to the plastic surface less tightly than do conventional biofilms.

These results show that, rather than being dependent on all six of the transcription factors regulating conventional biofilms, the pheromone-stimulated biofilms show dependencies on only four regulators (Bcr1, Tec1, Rob1, and Brg1; Figure 1C). One interpretation of this result is that because the pheromone-stimulated biofilms are less adherent they require only a subset of the conventional biofilm circuit. We note that deletion of *NDT80* shows opposite effects in conventional and pheromone-stimulated biofilms (deletion of *NDT80* compromises the former and enhances the latter, Figure 1D), and we return to this point later in the paper. Experiments in which a wild-type allele of the deleted gene was reintroduced into the homozygous deletion mutant significantly complemented all of the mutant phenotypes (Figure 1C and 1D). Complete complementation was not expected as these addback strains contained one functional gene copy compared to two gene copies in the wildtype strain.

Confocal images of pheromone-stimulated biofilms revealed them to be relatively patchy compared to conventional biofilms, although there was still a relevant correlation between CSLM images (Figure 1E and F) and measurements of adherent cells (Figure 1C and D). Having established the basic requirements for general biofilm production, we now turn to the components specific for pheromone-stimulated biofilm formation.

### Cph1 Is Essential for the Formation of Pheromone-Stimulated Biofilms

Previous studies have proposed that Tec1 is the master transcriptional regulator of pheromone-induced biofilms, while Cph1 is dispensable for their formation [14]. This result was surprising given that Cph1 or its orthologs are essential for pheromone signaling in multiple yeast species. We therefore directly compared the role of Cph1 and Tec1 in pheromone signaling in *C. albicans* white cells from multiple strain backgrounds.

**Figure 2:**
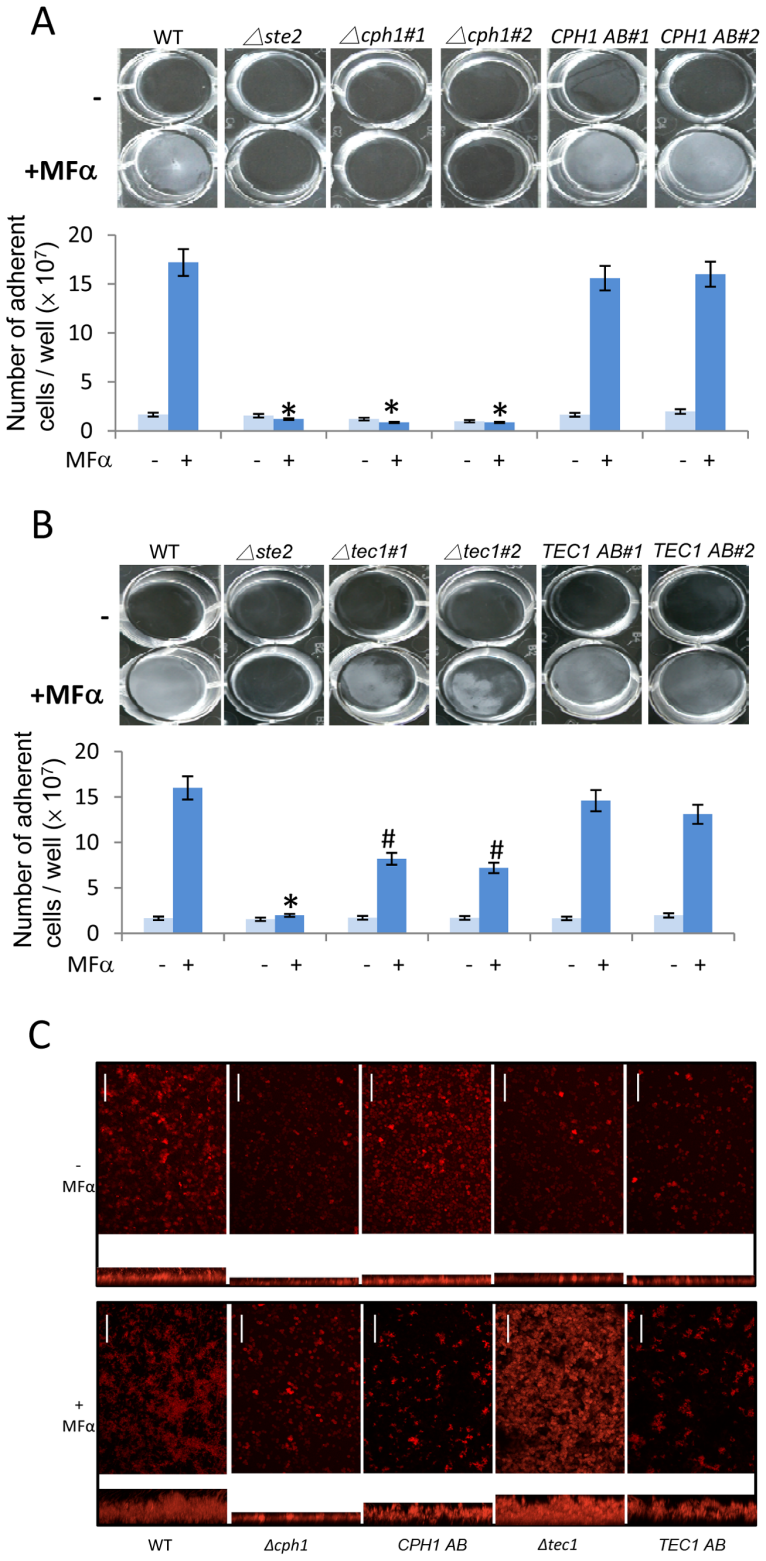
Role of Cph1 and Tec1 in the *C. albicans* response to pheromone by white cells. Pheromone-stimulated biofilm formation was measured in an adherence to plastic assay. P37005 cells (or mutant derivatives) were inoculated into 6-well cluster plates and treated with 10 µM α pheromone (MFα) for 24 h at room temperature. Wells were washed to remove non-adherent cells and photographed, or cells in the biofilm resuspended and quantified. (A) Cph1 is essential for pheromone-mediated biofilm formation. (B) Pheromone-stimulated biofilm formation is reduced, but not abolished, in the absence of Tec1. (C) Confocal scanning laser microscopy of biofilm formation also indicates that Cph1, but not Tec1, is necessary for biofilm formation in response to pheromone. For each image, the top panel shows the top view and the bottom panel shows the reconstructed side view, with the plastic substrate at the bottom of the image. Scale bars are 50 µm. AB indicates strains in which the target gene has been reintegrated into the mutant background. Values are the mean ± SD from two independent experiments with at least three replicates. “#” represents *P*<0.05 and “*” represents *P*<0.001 for WT v. mutant. Light blue bars on graphs, no pheromone added. Dark blue bars on graphs, pheromone present. (WT P37005: CAY716; *Δste2/Δste2*: CAY1234; *Δcph1/Δcph1*: CAY2899; *CPH1 AB*: CAY3028; *Δtec1/Δtec1*: CAY2506; *TEC1 AB*: CAY2750).

For these experiments, α pheromone was first used to stimulate biofilm formation in white cells of strain P37005 that is a natural *MTL* **a**/**a** isolate, and like SC5314 belongs to clade I, a major clade of *C. albicans* strains [23]. In contrast with published reports, we found that pheromone-stimulation of biofilm formation in white cells was strictly dependent on Cph1, as deletion of this factor abolished formation of biofilms (Figure 2A and C). Mutant strains missing Cph1 therefore resembled *Δste2/Δste2* mutants that are lacking the pheromone receptor and also failed to form biofilms (Figure 2A). Reintegration of the *CPH1* gene into *Δcph1/Δcph1* mutants restored biofilm formation close to wildtype levels, confirming the essential role of Cph1 in the white cell response to pheromone. To account for strain background differences, *cph1* deletion mutants were also constructed in SC5314 and these mutants were also found to be completely deficient in pheromone-stimulated biofilm formation (Figure S1). In contrast, loss of *CPH1* had no effect on conventional biofilm formation in either SC5314 or P37005 strain backgrounds (Figure S2).

We similarly re-examined the contribution of Tec1 to pheromone signaling in P37005 white cells. As shown in Figure 1, deletion of *TEC1* has a significant effect on both pheromone-stimulated and conventional biofilms in SC5314. This is also true in the P37005 background, as *tec1* mutants were defective in both models of biofilm formation (Figure 2B and Figure S2). However, unlike *cph1* mutants, pheromone treatment still promoted substantial biofilm formation in *tec1* mutants, while biofilm responses were abolished in the *cph1* strain. Together, these results indicate that Tec1 does not have a selective effect on pheromone-stimulated biofilms, but that it plays a general role in biofilm formation.

Pheromone-stimulated biofilms in P37005 were imaged by confocal scanning laser microscopy (CSLM). These assays demonstrated that biofilms were increased upon pheromone addition, with wildtype biofilms ∼125 µm in depth (Figure 2C). As expected, biofilms were greatly reduced in pheromone-treated *Δcph1/Δcph1* mutants (∼25 µm thick), whereas pheromone-treated *Δtec1/Δtec1* mutants produced a biofilm of intermediate thickness (∼100 µm) (Figure 2C). We note that pheromone-stimulated biofilms were substantially more fragile in the SC5314 strain background (Figure 1) compared to P37005 (Figure 2), and therefore more easily disturbed by washing. Nonetheless, we conclude that Cph1 is the master regulator of the white cell pheromone response in both SC5134 and P37005 strains of *C. albicans*. In contrast, Tec1 appears to play a more general role in biofilm formation and is not specifically required for the response to pheromone.

**Figure 3:**
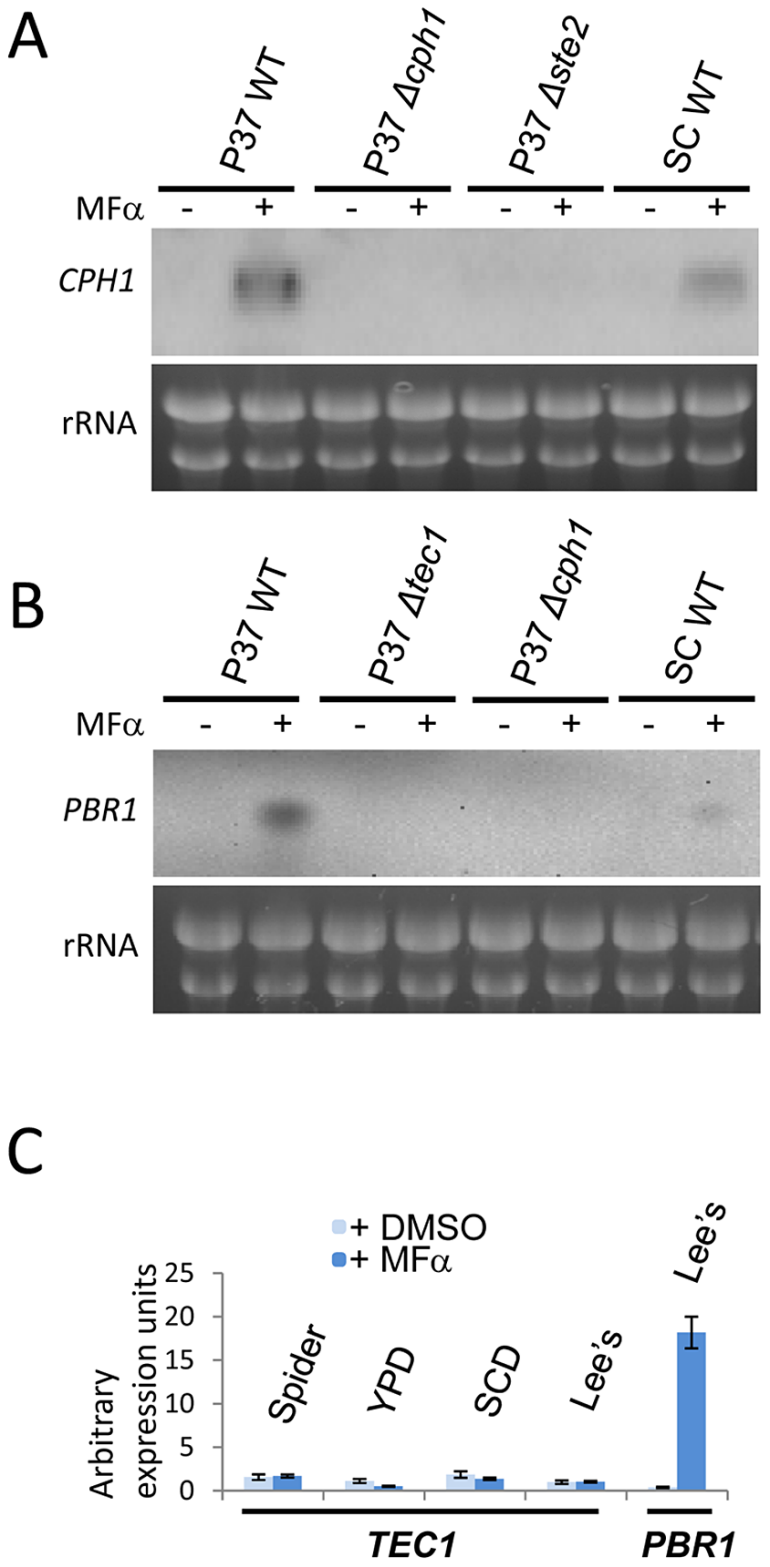
The *CPH1* gene is up-regulated in *C. albicans* white cells responding to pheromone. Northern blotting reveals that (A) *CPH1* is highly induced upon 10 µM α-factor treatment of P37005 white cells for 4 h in Spider medium. (B) *PBR1* is also highly induced in white cells responding to pheromone. (C) Quantitative RT-PCR indicated that expression of the *TEC1* gene did not change significantly following pheromone treatment under a variety of culture conditions. Each data is the mean ± SD from two independent experiments with at least three replicates. Light blue bars on graphs, no pheromone added. Dark blue bars, MFα pheromone present. (WT P37005: CAY716; *Δcph1/Δcph1*: CAY2899; *Δtec1/Δtec1*: CAY2506; *Δste2/Δste2*: CAY1234; WT SC5314: RBY717). P37 indicates strain derived from P37005, SC indicates derived from SC5314.

Transcriptional regulators at the bottom of a signaling cascade are often upregulated in response to the signal. To address whether *CPH1* or *TEC1* genes are induced upon pheromone treatment of white cells, northern analysis of gene expression was performed. Increased *CPH1* gene expression was observed in white cells of both P37005 and SC5314 strains when challenged with pheromone (Figure 3A). Expression of *PBR1*, a gene previously reported to be induced by α pheromone [15], was also increased in white cells treated with pheromone (Figure 3B), whereas *TEC1* expression was not detected by northern analysis (data not shown). Gene expression of *TEC1* and *PBR1* was also examined using quantitative RT-PCR. While *PBR1* was highly induced in white cells responding to pheromone (∼45-fold), *TEC1* expression levels were not induced by pheromone in any of the media conditions tested (Figure 3C). These results further support our finding that Cph1, and not Tec1, mediates transduction of the pheromone signal in *C. albicans* white cells.

### Mating of *C. albicans* Opaque Cells Is Mediated by Cph1

**Figure 4:**
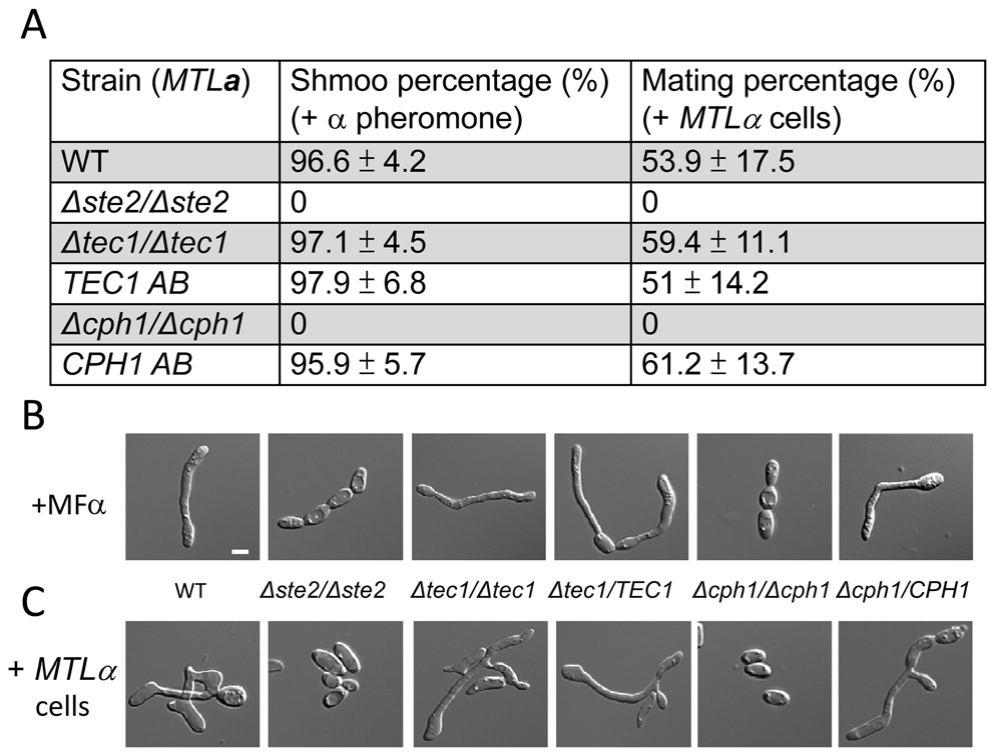
The formation of mating projections and a-α mating are mediated by the Cph1 transcription factor in opaque cells. (A) Deletion of *STE2* or *CPH1* abolished elongated projection formation and mating by *C. albicans* opaque cells. In contrast, *TEC1* is not required for pheromone responses in the opaque state. Numbers are the mean ± SD from two independent experiments with three replicates. Images of (B) mating projections formed in *MTL* **a** opaque cells responding to α pheromone, and (C) zygotes produced from mating between *MTL* **a** and *MTL*α opaque cells. Scale bar: 5 µm. Each data point is the mean ± SD from two independent experiments with at least three replicates. (WT: CAY1477; *Δste2/Δste2*: CAY1478; *Δtec1/Δtec1*: CAY2689; *TEC1 AB*: CAY2775; *Δcph1/Δcph1*: CAY2947; *CPH1 AB*: CAY3046; *MTL*α: DSY211).

In contrast to white cells, opaque cells efficiently upregulate the entire repertoire of mating genes and undergo **a**-α cell fusion in response to pheromone. We addressed the roles of Cph1 and Tec1 in opaque cell signaling by quantifying morphological responses (elongated projections) in response to pheromone as well as mating frequencies, under standard (non-biofilm) conditions. Consistent with previous reports [14], [17], [18], Cph1 was essential for the pheromone response in opaque cells, as *Δcph1/Δcph1* mutants lacked detectable projection formation and did not undergo **a**-α mating (Figure 4). Reintegration of the *CPH1* gene into the mutant strain restored these phenotypes to wildtype levels. In contrast, *Δtec1/Δtec1* mutants displayed normal mating projection formation (97%) when challenged with α pheromone, as well as normal **a**-α mating efficiency (59%) (Figure 4). These results establish Cph1 as the master regulator of pheromone signaling in both white and opaque cells of *C. albicans*.

### Gene Expression Profiles of Pheromone-Responding White Cells

Transcriptional profiling of the pheromone-stimulated response previously showed that more than 300 genes are induced in *C. albicans* opaque cells, while only 30 genes are induced in white cells [6], [24]. These responses are media dependent, with opaque cells exhibiting the strongest response in Spider medium, while white cells are most responsive in Lee's medium [24]. In addition, previous profiling experiments were performed under planktonic conditions and cellular responses in biofilms were not examined.

**Figure 5:**
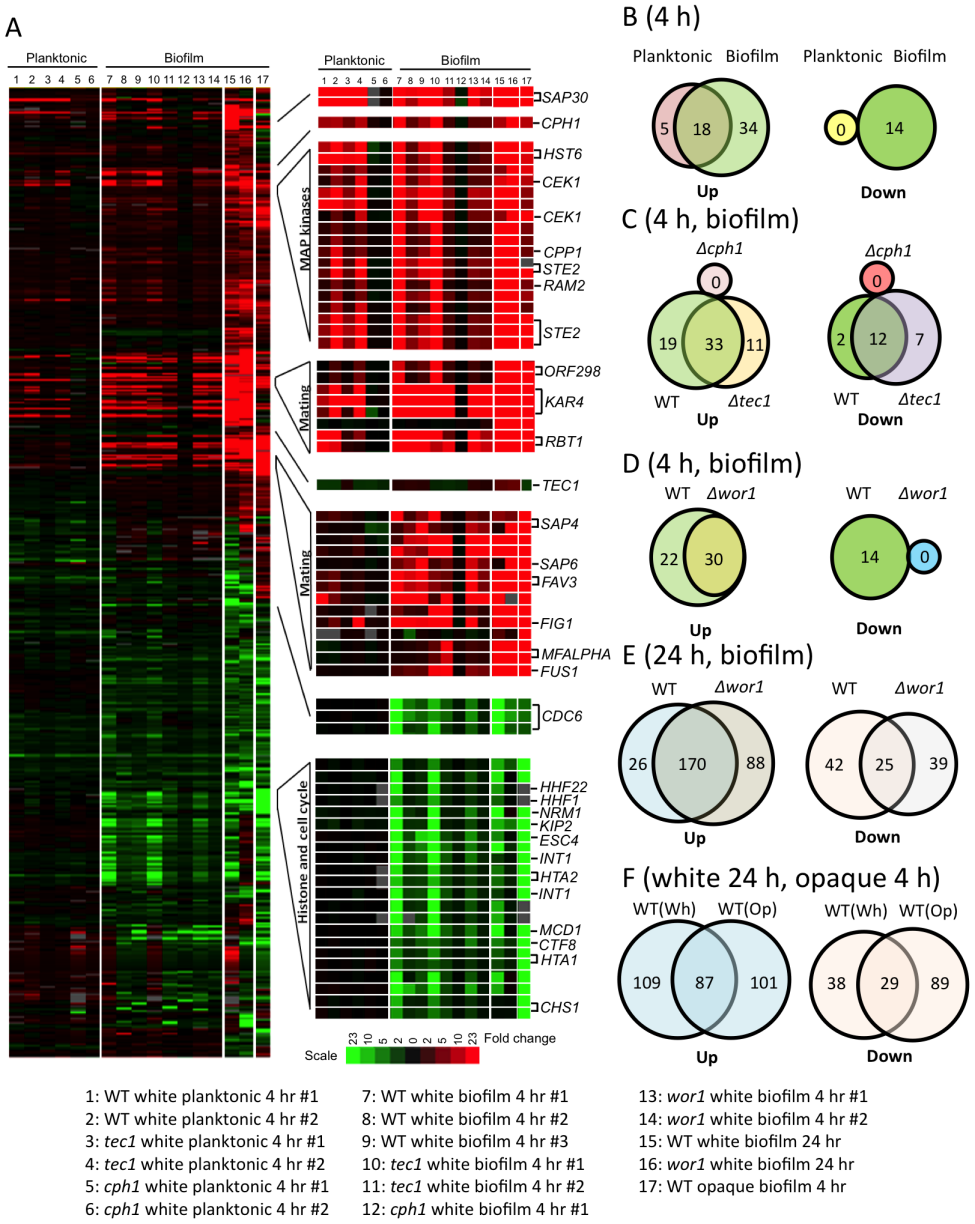
Transcriptional profiling of *C. albicans* white and opaque cells in response to pheromone. *C. albicans* P37005 cells were grown in Lee's medium at 25°C as planktonic cells or under biofilm conditions. Cells were treated with 10 µM α pheromone or a DMSO mock control, and collected after incubation for 4 h or 24 h. cDNA was prepared and hybridized against a *C. albicans* Agilent microarray (see Materials and Methods). For each array, pheromone-treated samples were hybridized against the mock-treated control. (A) Left panel: gene expression of *C. albicans* white (lanes 1–16) and opaque (lane 17) cells in planktonic (lane 1–6) or biofilm (lane 7–17) culture conditions. Pheromone up-regulated genes are shown in red, and down-regulated genes are shown in green. Profiling reveals that Cph1 (lanes 5–6 and lane 12) is essential for pheromone signaling in *C. albicans* white cells under both planktonic and biofilm conditions. Fold changes in gene expression are presented in Table S3. (B) Comparison of numbers of genes up- or down-regulated between different planktonic and biofilm culture conditions (lanes 1–2 vs. lanes 7–9). (C) Difference in gene expression between WT, *cph1* and *tec1* strains under biofilm conditions (lanes 7–9 vs. lanes 10–11 vs. lane 12). (D) Comparison between gene expression in WT and *wor1* strains (lanes 7–9 vs. lanes 13–14) in biofilm conditions at 4 h. (E) Changes of gene expressions between the WT and *wor1* mutant after pheromone treatment for 24 h (lane 15 vs. lane 16). (F) Comparison of pheromone-stimulated white (Wh, 24 h induction) and opaque (O, 4 h induction) gene expression under biofilm culture conditions (lane 15 vs. 17). (B–F) In all venn diagrams where there is overlap, the overlap is significant by a chi squared test (p<5×10^−254^).

To determine the gene expression profile of white cells undergoing pheromone-stimulated biofilm formation, we performed profiling of P37005 cells induced to adhere to the plastic surface or grown under planktonic conditions (Figure 5). For each data point, the transcriptional response in the presence of pheromone was compared to the response in mock-treated controls. White cells examined under both planktonic and biofilm conditions showed pheromone-induced expression of many genes related to mating and pheromone MAPK signaling (Figure 5A, lanes 1–2 and 7–9, Figure 5B, and Table S3). Thus, despite the fact that white cells are mating incompetent, genes involved in pheromone sensing (*STE2*), pheromone secretion (*HST6*), and pheromone modification (*RAM2*) are upregulated, as well as genes associated with mating and karyogamy (*FIG1*, *FUS1*, and *KAR4*). Biofilm conditions resulted in an enhanced response to pheromone; 52 genes were induced by pheromone after 4 hours in biofilm conditions compared to 23 genes under planktonic conditions (Figure 5B). Multiple genes were also repressed under biofilm conditions (14 genes) while no genes were repressed >4 fold in planktonic conditions, and repressed genes were associated with DNA replication and the cell cycle (Figure 5B). Many of the ‘biofilm-specific’ genes were also expressed under planktonic conditions but did not pass the 4-fold cutoff (data not shown). Overall, the data indicates that the transcriptional response to pheromone in planktonic cells is primarily a subset of the response under biofilm conditions. These findings establish that the mode of growth, in addition to the culture medium, can markedly influence the strength of the transcriptional response to environmental signals.

We similarly performed profiling on *Δcph1/Δcph1* and *Δtec1/Δtec1* mutants to determine the contribution of these factors to the transcriptional response to pheromone in white cells (Figure 5A, lanes 3–6 and 10–12). Notably, loss of *CPH1* essentially abolished the entire transcriptional response to pheromone under both planktonic and biofilm conditions (lanes 5, 6, and 12). In contrast, deletion of *TEC1* only slightly compromised the transcriptional response, as 44 genes were induced in *Δtec1/Δtec1* cells compared to 52 genes in the wildtype strain (Figure 5A, lanes 3–4 and 10–11, and Figure 5C). Consistent with our northern blot and RT-PCR data, *CPH1* was itself induced (∼7-fold) in white cells exposed to pheromone, whereas the *TEC1* transcript was not induced at 4 hours and was only weakly induced (<3-fold) at 24 hours (Figure 5A and Table S3). These observations support our conclusion that Cph1, and not Tec1, is the transcriptional mediator of the white response to pheromone.

The switch between white and opaque forms occurs approximately once every 10^4^ generations, although switching is also highly dependent on environmental factors [25]–[29]. To ensure that our profiling analysis accurately reflects gene expression from white cells and not from a contaminating minority of opaque cells, we also performed profiling on cells locked in the white developmental state. To this end, a *Δwor1/Δwor1* mutant was constructed in the P37005 strain background, as loss of *WOR1* prevents cells switching from white to opaque [30]–[32]. Expression profiles of wildtype and *Δwor1/Δwor1* strains were similar, with pheromone signaling components and mating genes induced in both strains (Figure 5A and 5D, and Table S3). While most profiling experiments were performed at 4 hours post-pheromone treatment, expression profiles were also compared at 24 hours following pheromone addition in wildtype and “white-locked” cells (Figure 5A, lanes 15 and 16, respectively). Gene induction was increased in wildtype (196 genes) and *Δwor1/Δwor1* (258 genes) strains at the 24-hour time point (Figure 5E and Table S3). This data reveals that white cells can mount a substantial response to pheromone exposure, and that the response is significantly stronger at 24 hours than at 4 hours.

Finally, the transcriptional response to pheromone was compared between white and opaque cells, both grown under biofilm conditions (Figure 5A, lanes 15 and 17, and 5F). We note that opaque cells form very weakly adherent pheromone-induced biofilms under these assay conditions [9], [33]. Overall, the number of the genes induced in opaque cells at 4 hours (188 genes) was similar to the number induced in white cells at 24 hours (196 genes, see Table S3). These results indicate that opaque cells in biofilms are generally more responsive to pheromone challenge than white cells and are consistent with previous results obtained under planktonic conditions [24]. Many of the genes induced in white and opaque biofilms were shared (87 genes), and this overlap was significant (p<5×10^−254^) (Figure 5F). With a 4-fold cutoff, it appears that 109 genes were induced only in white cells and 101 genes were induced only in opaque cells, however, a number of these genes were induced at least 2-fold in both white and opaque cells. After removal of these genes, there remain 76 white-specific and 59 opaque-specific genes, indicating that there is a unique transcriptional program acting in each cell type. A comparative table showing genes regulated by pheromone in white and opaque biofilms is provided (Table S4). Overall, our data indicates that phase-specific genes may play an important role in the different phenotypic outputs of pheromone signaling in *C. albicans*; biofilm formation in white cells and mating in opaque cells.

We also compared the transcriptional program in pheromone-induced biofilms to that recently described in conventional biofilms [13]. Using a 2-fold cutoff for up- and down- regulated genes, we found that 662 genes were induced in conventional biofilms, while 486 genes were induced in pheromone-induced biofilms in white cells (Figure S3). 128 genes were shared between these two transcriptional programs (p = 2×10^−30^). The significance of this overlap is lost when using a more stringent cutoff (p = 0.3 for a 4-fold cutoff). Similarly, gene overlap between repressed genes in the two biofilm models was significant using a 2-fold cutoff (p = 9×10^−3^), but not when using a 4-fold cutoff (p = 0.6) (Figure S3). Gene Ontology analysis revealed that the 128 genes upregulated more than 2-fold in both datasets are enriched for genes involved in adhesion (p<1×10^−5^), including *HWP1*, *HXK1*, *XOG1*, *SUN41*, *PHR1*, *RFX2*, *SAP4*, *SAP5*, *SAP6*, *ALS1*, *TEC1* and *PBR1*. These results indicate that the transcriptional changes occurring during conventional and pheromone-induced biofilms are partially overlapping, but that the genes undergoing the highest fold changes in transcription are generally unique to each program.

### Identification of *HGC1*, a Downstream Target of Cph1 and Tec1, for Pheromone-Stimulated Biofilm Formation

**Figure 6:**
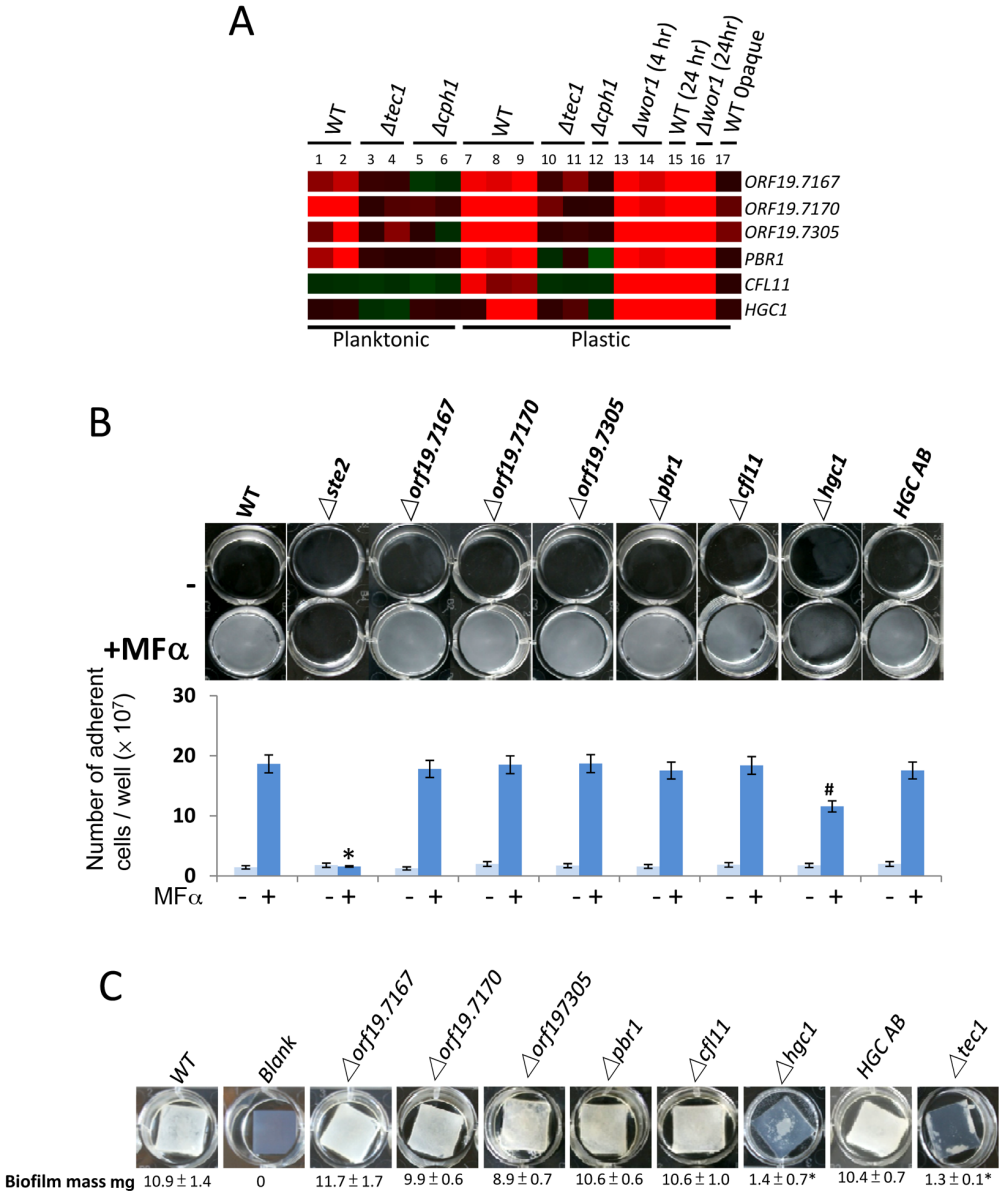
Examination of six downstream targets of Cph1 and Tec1 reveals a general role for Hgc1 in biofilm formation. (A) Heatmap showing expression of six target genes that are regulated by Cph1 and Tec1 during pheromone treatment of white cells. (B) Pheromone-stimulated biofilm formation in mutants lacking each of the six candidate genes. Top panel, images of white cells adhering to plastic. Bottom panel, quantification of the number of adherent cells. Light blue bars on graphs, no pheromone added. Dark blue bars, MFα pheromone present. (C) Analysis of the six candidate genes in a conventional biofilm assay on silicone squares. Hgc1 and Tec1 are both necessary for conventional biofilm formation. Values are the mean ± SD from two independent experiments with at least three replicates. “#” represents *P*<0.01 and “*” represents *P*<0.001 for the difference with the wildtype strain. The complemented *HGC1* strain (HGC AB) showed a significant increase in biofilm formation compared to the *hgc1* mutant. (WT: CAY716; *Δste2/Δste2*: CAY1234; *Δorf19.7167/Δorf19.7167*: CAY3445; *Δorf19.7170/Δorf19.7170*: CAY3447; *Δorf19.7305/Δorf19.7305*: CAY3693; *Δpbr1/Δpbr1*: CAY3689; *Δcfl11/Δcfl11*: CAY3687; *Δhgc1/Δhgc1*: CAY3465; *HGC1 AB*: CAY3702).

Transcriptional profiling of wildtype, *Δcph1/Δcph1* and *Δtec1/Δtec1* strains revealed a number of potential downstream targets of Cph1 and Tec1. In total, we observed 13 genes that exhibited decreased induction by pheromone (>2-fold) in *cph1* and *tec1* mutants (Table S3 and data not shown). Of these genes, six candidates were chosen for further analysis due to their dependence on *CPH1* and *TEC1* for pheromone-induced expression, and also because they were not induced in pheromone-treated opaque cells (Figure 6A). The lack of induction in mutant white strains suggested these genes may play important roles in biofilm development in *C. albicans*.

The six candidate genes were *PBR1*, *CFL11*, *HGC1*, *ORF19.7167*, *ORF19.7170*, and *ORF19.7305*. *PBR1* has previously been implicated in pheromone-stimulated biofilms [15], *CFL11* is induced during the early development of conventional biofilms [34], and *HGC1* is a G1 cyclin-related protein required for hyphal formation and virulence [35]. Little is known about *ORF19.7167*, *ORF19.7170* and *ORF19.7305*, although *ORF19.7167* is a predicted adhesin-like protein [36]. Each of the six candidate genes were deleted in the P37005 strain background and tested for pheromone-stimulated biofilm formation (Figure 6B). Only the *Δhgc1/Δhgc1* mutant showed a significant reduction in biofilm formation when challenged with pheromone, while loss of the other five genes did not impact biofilm formation. Adding a functional copy of *HGC1* back into the mutant strain restored biofilm formation, confirming that this gene is necessary for efficient pheromone-induced biofilm formation (Figure 6B).

We also tested the role of the six candidate genes in conventional biofilm development. Once again, only deletion of *HGC1* had a significant effect on biofilm formation (Figure 6C). Thus, while the average mass of wildtype biofilms was ∼11 µg, biofilms formed by the *Δhgc1/Δhgc1* mutant were only ∼1 µg. Given the key role of Hgc1 in hyphal formation [35], it is likely that the reduced filamentation of *hgc1* mutants compromises their ability to form conventional biofilms. However, Hgc1 may play additional roles in adherence and/or biofilm maturation in light of *CPH1*-dependent upregulation of Hgc1 in pheromone-stimulated biofilms.

The role of Hgc1 in sexual mating was also addressed. Opaque **a** strains lacking *HGC1* were found to undergo efficient sexual mating with a wildtype α partner (Figure S4A). The response to α pheromone was also normal in mutant *hgc1* **a** cells (Figure S4A and B). These experiments indicate that, in contrast to Cph1, Hgc1 does not have a detectable role in the mating program.

Taken together, these results identify Hgc1 as an important regulator of biofilm formation in both conventional and pheromone-stimulated biofilm models, but that this factor is dispensable for mating. Surprisingly, we note that deletion of *PBR1* did not alter biofilm formation in white cells responding to pheromone, in contrast with a previous report [15]. Deletion of *PBR1* also did not have a visible effect on conventional biofilm formation in our assays.

### A Cell Separation Defect Promotes Pheromone-Stimulated Biofilms

**Figure 7:**
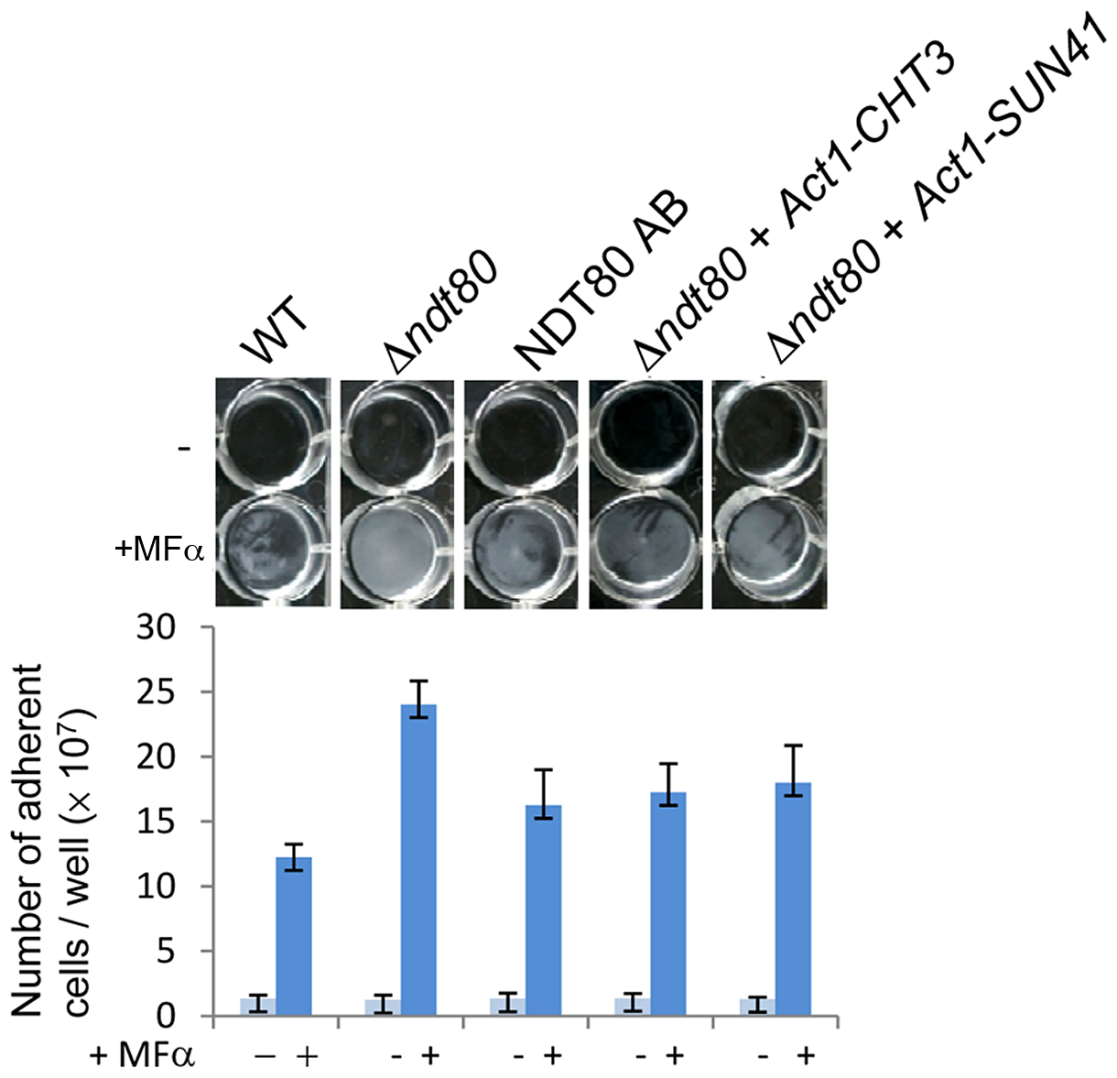
Role of *NDT80* in formation of pheromone-stimulated biofilms. White cells lacking *NDT80* formed hyper-biofilms that contained increased numbers of adherent cells and were difficult to remove from the plate. Overexpression of cell wall degrading genes, *CHT3* or *SUN41*, decreased hyper-biofilm formation in the *ndt80* background. Each data point is the mean ± SD from two independent experiments with at least three replicates. “*” represents *P*<0.001 for WT v. *Δndt80*. “#” represents *P*<0.05 for *Δndt80* v. *Δndt80 AB*, *Δndt80 v. Δndt80+Act1-CHT3*, and *Δndt80* v. *Δndt80+Act1-SUN41*. (WT: RBY717; *Δndt80*: RBY520; *NDT80 AB*: CAY3593; *Δndt80*-Act1-CHT3: CAY3818; *Δndt80*-Act1-SUN41: CAY3816).

One of the most surprising aspects of our comparison of conventional and pheromone-stimulated biofilms is the role of the transcription regulator Ndt80. Ndt80 is required for conventional biofilms (Figure 1 and [13]), yet its deletion results in increased pheromone-stimulated biofilm formation (Figure 7). Ndt80 plays a pleiotropic role in *C. albicans* including regulation of the glycosidase gene *SUN41* and the endochitinase gene *CHT3* [37]. In the absence of *NDT80*, expression of *SUN41* and *CHT3* genes is compromised, leading to a defect in cell separation and growth as chains of cells [37]. We therefore tested whether enhanced biofilm formation in the *ndt80* mutant was due, at least in part, to its cell separation defect. To examine this possibility, overexpression of cell wall degradation genes was carried out in the *ndt80* background. Indeed, overexpression of either *SUN41* or *CHT3* in the *ndt80* mutant resulted in a ∼30% reduction in biofilm formation (Figure 7). These results indicate that increased formation of pheromone-stimulated biofilms in *ndt80* mutant strains can be attributed, at least in part, to a defect in cell separation.

## Discussion

Biofilms represent structured communities of cells that are of clinical importance for human pathogens such as *C. albicans*. They exhibit increased drug resistance relative to planktonic cells and can seed bloodstream infections that result in life-threatening systemic disease [10], [12]. “Conventional” *C. albicans* biofilms have been extensively studied and involve the adherence of yeast cells to a surface, followed by maturation of the biofilm by filamentous growth and production of the extracellular matrix. However, when grown under a different set of culture conditions a distinct type of biofilm is formed. Unlike conventional biofilms, formation of this alternative biofilm is stimulated by mating pheromone [9]. In this paper, we have compared the genetic requirements and transcriptional control of conventional and pheromone-stimulated biofilms.

### Cph1 Is the Key Transcription Factor Regulating Pheromone Signaling in *C. albicans*

**Figure 8:**
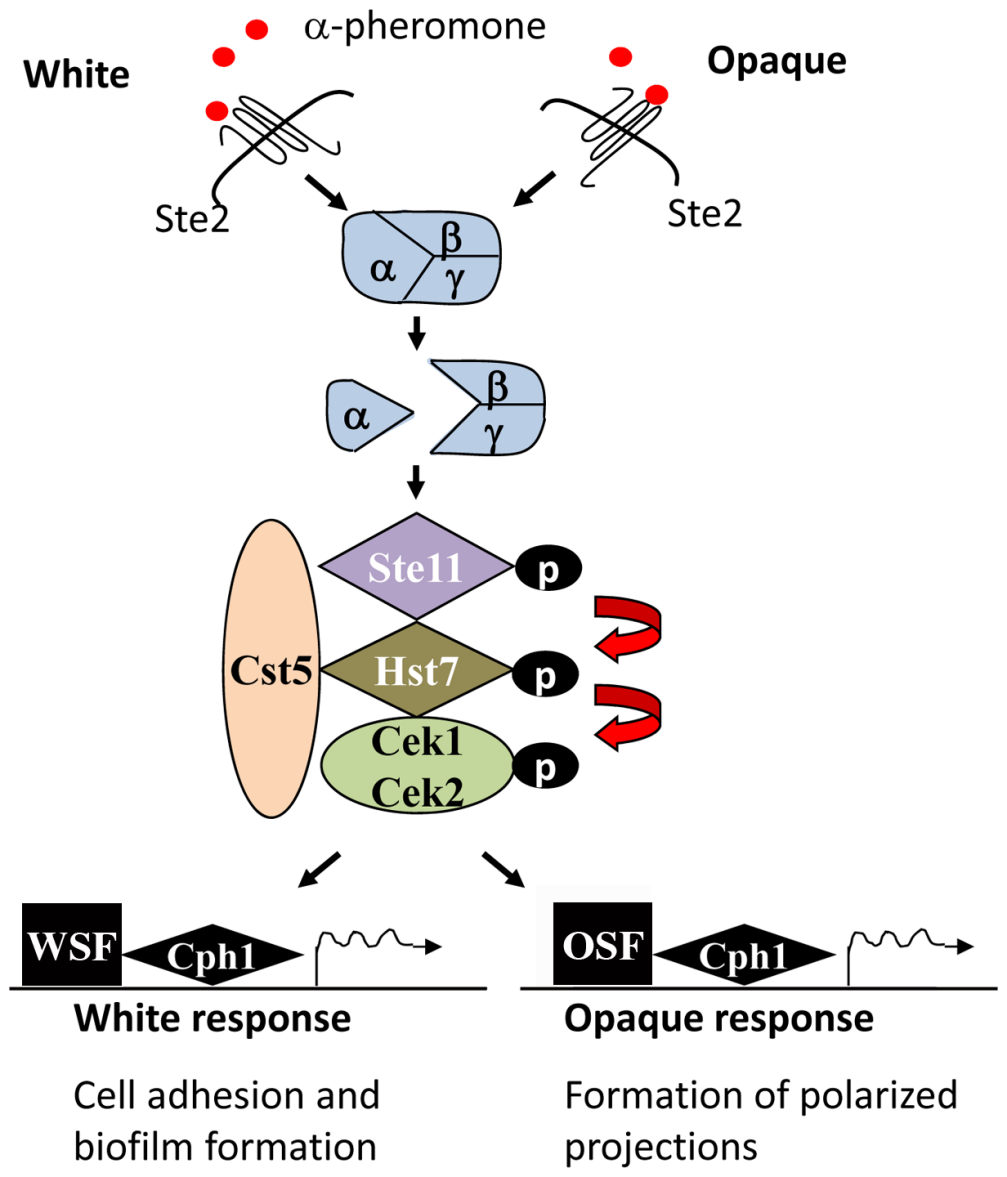
Model for pheromone signaling responses in *C. albicans* white and opaque cells. The same pheromone MAP kinase pathway operates in *C. albicans* white and opaque cells, and activates downstream responses via the same transcription factor, Cph1. White- and opaque-specific factors (WSF and OSF, respectively) work in concert with Cph1 to drive phase-specific phenotypes. The interactions with Cph1 could either be direct or indirect. In response to pheromone, *C. albicans* opaque cells undergo efficient polarized growth and cell conjugation. In contrast, white cells cannot mate but can form pheromone-stimulated biofilms under certain environmental conditions.

We demonstrate that the transcription factor Cph1 (ortholog of *S. cerevisiae* Ste12) is the master regulator of pheromone signaling in *C. albicans*, as deletion of this gene abolished both pheromone-stimulated biofilms by white cells and sexual mating by opaque cells. This result was surprising, as previous reports had indicated that Cph1 was critical for the opaque response to pheromone but dispensable for the white response [19]. Instead, the Tec1 transcription factor was proposed to be the downstream target of the pheromone MAP kinase cascade in white cells [14], [38]. These studies led to a model of pheromone signaling whereby Cph1 directed mating gene expression in opaque cells, while Tec1 regulated biofilm formation in opaque cells [14], [38]. On the basis of the results described here, we propose a new model in which the same MAP kinase components and Cph1 transcription factor are responsible for signal transduction in both white and opaque cells (Figure 8).

Consistent with Cph1, and not Tec1, mediating white cell signaling, the *CPH1* gene was highly induced by pheromone under both planktonic and biofilm culture conditions. In contrast, *TEC1* was only weakly induced after 24 h in pheromone-induced biofilms. Deletion of Cph1 also abolished the genome-wide transcriptional response to pheromone in white cells and completely inhibited pheromone-induced biofilm formation. By comparison, loss of Tec1 resulted in the altered expression of only a subset of pheromone-induced genes and pheromone treatment still significantly enhanced biofilm formation in the *tec1* mutant. Moreover, deletion of Tec1 compromised biofilm formation under both conventional and pheromone-stimulated conditions, indicating that Tec1 has a general effect on biofilm formation that is not specific to pheromone stimulation. Results with Cph1 and Tec1 were similar when compared between *C. albicans* P37005 and SC5314 strains, confirming that mutant phenotypes were similar in different strain backgrounds.

### Identification of Downstream Targets of Cph1

Transcriptional profiling was used to uncover factors that act downstream of Cph1 (either directly or indirectly) in biofilm formation. Six genes were identified that are pheromone-induced in wildtype white cells under biofilm conditions but are not induced in either *tec1* or *cph1* mutants (Figure 6). Several of these candidates had previously been implicated in cell adhesion and/or biofilm formation [15], [34]–[36]. Deletion of five of the six genes, however, failed to result in a defect in pheromone-stimulated biofilm formation. Either these genes do not play a role in biofilm development or their roles are masked by redundancy with other genes. Functional redundancy has been observed in other biofilm studies so that deletion of multiple factors is often necessary to observe a biofilm defect [39], [40]. However, it was surprising that loss of *PBR1* (*P*heromone stimulated *B*iofilm *R*egulator 1) did not affect biofilm development in any of our assays, as it was previously reported to be critical for this process [15].

One gene product shown to significantly influence biofilm formation was Hgc1. Deletion of *HGC1* resulted in decreased biofilm formation in white cells responding to pheromone, and also abolished formation of conventional biofilms. Hgc1 is therefore important for cell adhesion and biofilm development in both models of biofilm formation. Hgc1 is a G1 cyclin-related protein involved in hyphal morphogenesis and virulence [35], [41]. Cells lacking Hgc1 exhibit a marked defect in hyphal formation, which may explain the inability of *hgc1* mutants to form conventional biofilms. Presumably, Hgc1 also contributes to biofilm formation by other mechanisms, as hyphal growth was rarely observed in pheromone-stimulated biofilms and is unlikely to play an important role in this process.

### Transcriptional Regulation of Conventional and Pheromone-Stimulated Biofilms

Recent studies have identified the core transcriptional network regulating conventional biofilm formation. Loss of any one of six transcription factors (*BCR1*, *BRG1*, *EFG1*, *NDT80*, *ROB1*, or *TEC1*) compromised biofilm formation both *in vitro* and *in vivo* [13]. The transcriptional changes during conventional and pheromone-induced biofilms show partial overlap (Figure S3), and we found that mutants lacking Bcr1, Brg1, or Rob1, in addition to Tec1, were also deficient in pheromone-stimulated biofilm formation. Four of the six master transcription factors therefore play a general role in mediating cell adherence and/or biofilm maturation.

Surprisingly, although *NDT80* is necessary for conventional biofilm formation, we observed that loss of *NDT80* resulted in significantly thicker pheromone-induced biofilms than those formed by wildtype cells. Previous studies have established that *C. albicans* Ndt80 plays diverse roles in drug resistance and conventional biofilm formation [13], [37], [42], and is also required for expression of cell separation genes (e.g., *SUN41* and *CHT3*) whose gene products enable the separation of mother and daughter cells [37], [43]. We found that pheromone-induced hyper-biofilm formation in the *ndt80* mutant was significantly suppressed by overexpression of *SUN41* or *CHT3*. Our results therefore demonstrate that the cell separation defect in *ndt80* mutants contributes to the formation of hyper-biofilms. Moreover, regulation of cell separation could play a general role in fungal biofilm formation, either by promoting the aggregation of cells within a biofilm or by increasing cell accumulation on the substrate surface.

### Profiling of Pheromone-Stimulated Biofilms Reveals Both Phase- and Biofilm-Specific Factors

Despite utilizing the same signaling cascade, white and opaque cells exhibit very distinct phenotypes upon pheromone challenge. To dissect these differences, we performed transcriptional profiling on pheromone-treated white and opaque cells under both planktonic and biofilm conditions. White and opaque cells exhibited significant overlap in their transcriptional responses, although the overall response was weaker in white cells than in opaque cells. In fact, the response at 24 hours in white cells was closest to that at 4 hours in opaque cells (Figure 5F). We also note that differences in gene expression were observed between pheromone-treated cells under planktonic and biofilm conditions. In general, pheromone responses were stronger under biofilm conditions, with increased expression of mating genes and stronger inhibition of DNA replication and cell cycle genes. These results establish that planktonic and biofilm cells experience different microenvironments with direct consequences for gene expression and function.

### Pheromone Signaling and the White-Opaque Switch

Given that Cph1 mediates pheromone signaling in both white and opaque states, how do these cell types produce distinct biological outputs? Presumably, white and opaque specific components are responsible, at least in part, for mediating these different responses. In *S. cerevisiae*, Ste12 (the Cph1 ortholog) can activate different signaling pathways through selective interactions with different transcription factors [44], [45]. Pheromone signaling induces Ste12 homodimers that induce expression of mating genes, whereas a Ste12/Tec1 complex mediates activation of filamentous growth [44], [45]. By analogy, it is possible that *C. albicans* Cph1 cooperates with different co-factors in white and opaque cells, thereby directing biofilm formation and sexual mating, respectively (Figure 8). In support of this model, different subsets of genes were induced by pheromone in white and opaque cells, indicating transcriptional activation of distinct pathways.

White and opaque cells may also exhibit differences in biofilm formation due to inherent structural differences. In addition to differences in cell shape, white and opaque states exhibit marked differences in cell wall morphology, phase-specific antigens, and actin motility [46], [47]. Additional studies will now be necessary to further characterize the physical differences between white and opaque cells and to reveal the roles of these two cell types in biofilm proficiency.

Finally, we note that studies in related species will also help shed light on the mechanisms regulating white- and opaque-specific responses. The white-opaque switch has been described in the related species *C. dubliniensis* and *C. tropicalis*, where opaque cells again represent the mating-competent form [48], [49]. It is therefore likely that the white-opaque switch evolved in the ancestor to *C. albicans*, *C. dubliniensis* and *C. tropicalis*. Future studies will compare white and opaque responses in these related pathogens to determine if mechanisms of pheromone signaling have been conserved between species, or if they have accrued different functions since they last shared a common ancestor. These approaches will further define the properties of each cell type that underlie the ability to generate distinct phenotypic outputs.

## Materials and Methods

### Media and Regents

Media and pheromone used in these experiments were prepared as described previously [50]–[52].

### Plasmid and Strain Construction

*C. albicans* strains and oligonucleotides used in this study are listed in Tables S1 and S2, respectively. To generate *Δtec1/Δtec1* strains, the 5′ flanking and 3′ flanking regions of *TEC1* were PCR amplified using primers 1167/1168 and 1169/1170, respectively. The 5′ and 3′ PCR products were digested with *Apa*I/*Xho*I and *Sac*I/*Sac*II, respectively, and cloned into the plasmid pSFS2a [53] to generate the plasmid pSFS-Tec1 KO. The plasmid was digested with *Apa*I/*Sac*I and transformed into either P37005 or an *MTL* **a**/**a** derivative of SC5314 (CAY716 or RBY717) to generate heterozygous *Δtec1/TEC1* mutants. The *SAT1* marker was recycled [54] and the strains re-transformed with the deletion construct to generate *Δtec1/Δtec1* strains CAY2506 and CAY2504. A *TEC1* complementation construct was made by amplification of the promoter and ORF using oligos 1334/1335. The PCR product was digested with *Apa*I/*Xho*I and cloned into pSFS2a to generate pSFS2a-TEC1 AB. The plasmid was linearized with *EcoR*I and transformed into CAY2504 and CAY2506 to create CAY2748 and CAY2750, respectively.

For *cph1* mutants, primers 1336/1337 and 1338/1346 were used to amplify the 5′ and 3′ regions of the *CPH1* gene. 5′ and 3′ PCR products were digested with *Kpn*I/*Apa*I and *Sac*I/*Sac*II and cloned into pSFS2a to generate pSFS2a-Cph1 KO. The construct was linearized with *Kpn*I/*Sac*I and transformed into CAY716 and RBY717 to generate heterozygous deletions. The *SAT1* marker was recycled and strains again transformed with the deletion construct to generate *Δcph1/Δcph1* strains CAY2899 and CAY2895, respectively. The *CPH1* complementation plasmid was constructed by PCR using primers 1419/1420, and the PCR fragment digested with *Not*I/*Sac*I and cloned into pSFS2a to create pSFS-CPH1 AB. The construct was then digested with *Hpa*I and transformed into CAY2899 and CAY2895 to generate CAY3025 and CAY3028, respectively.

To generate gene deletions of *ORF19.7167*, *ORF19.7170*, *ORF19.7305*, *PBR1*, *CFL11* and *HGC1*, 5′ and 3′ flanking regions of each gene were amplified using primers 1226/1227 and 1228/1229, 1194/1195 and 1196/1197, 1187/1188 and 1189/2115, 1218/1219 and 1220/1221, 1202/1203 and 1204/1205, 1210/1211 and 1212/1213, respectively. The PCR products were digested with *Apa*I/*Xho*I and *Sac*I/*Sac*II and cloned into pSFS2a to generate pSFS-7167 KO, pSFS-7170 KO, pSFS-7305 KO, pSFS-PBR1 KO, pSFS-CFL11 KO and pSFS-HGC1 KO, respectively. These constructs were linearized with *Apa*I/*Sac*I and transformed into CAY716 to generate CAY3445, CAY3447, CAY3693, CAY3689, CAY3687 and CAY3465, respectively. The *HGC1* complementation plasmid was constructed by PCR using primers 1845/1875 to amplify the promoter and ORF of *HGC1*. The PCR product was cloned into pSFS2a using *Apa*I/*Xho*I to generate pSFS2a-HGC1 AB. This construct was linearized with *Sna*I and transformed into CAY3488 to create CAY3702.

Gene deletions of *ROB1*, *BRG1*, and *BCR1* were achieved using an established fusion PCR approach [55]. 5′ and 3′ ORF flanking regions for *ROB1*, *BRG1*, and *BCR1* were amplified using oligos 1773/1774, 1775/1776, 1781/1782, 1783/1784, 1765/1766, and 1767/1768, respectively. These PCR products were then combined with a selectable marker (*HIS1* or *LEU2*) by fusion PCR, as described [55]. Fusion PCRs were used to delete target genes in RBY1132 to generate homozygous deletions in *ROB1* (CAY3670), *BRG1* (CAY3583) and *BCR1* (CAY3672).

For the *ROB1* gene addback construct, a PCR fragment was amplified using primers 1875/1876, digested with *Apa*I/*Sal*I and cloned into pSFS2a to generate pSFS-Rob1 AB. The construct was then linearized with *Apa*BI and transformed into CAY3670 to create CAY3805. *BCR1* and *BRG1* complementation plasmids were cloned using primers 1878/1879 and 1881/1882, respectively. PCR products were digested with *Apa*I/*Xho*I and cloned into pSFS2a to generate pSFS-BCR1 AB and pSFS-BRG1 AB, respectively. The constructs were digested with *Eco*RI and *Bam*HI and transformed into CAY3672 and CAY3583 to create CAY3858 and CAY3802, respectively.

To delete the *MTL*α locus and generate *MTL* **a**-type cells, plasmid pJD1 (GenBank accession #JX486681) was digested with *Xma*I, and transformed into OHY13, TF22, TF95, TF110, TF115, TF137, and TF156 to create OHY13a, TF22a, TF95a, TF110a, TF115a, TF137a, and TF156a, respectively. pJD1 was created using primers MBL 660/661 (3′ flank) and MBL 662/663 (5′ flank) to amplify ∼500 bp regions flanking the *MTL* loci such that the regions were homologous to both *MTL* **a** and *MTL*α. The flanking regions were fused to the *Candida dubliniensis ARG4* marker, which was amplified with primers UP2 and UP5 from pSN69 [56], using primers MBL 661/663 that introduced the *Xma*I site to each end of the fusion product. The amplified product was digested with *Xma*I and ligated into pUC19 (New England Biolabs).

### Pheromone-Stimulated Biofilm Assays

*C. albicans* white cells were grown in Spider medium at room temperature overnight. 5×10^7^ cells were added to 12 well dishes (Costar, Corning Inc.) and mixed with 1 ml Lee's medium [51] in the presence of 0.01% DMSO or 10 µM *C. albicans* synthetic α pheromone. Cultures were mixed and incubated without shaking at room temperature for 24 h. Supernatants were removed and wells washed with phosphate-buffered saline (PBS) and photographed. Each experiment was performed using at least two independent isolates with three experimental replicates.

### Conventional Biofilm Assays

We used a previously established protocol to measure dry weight of biofilms in a silicone model of conventional biofilm formation [55]. Pre-weighed sterile silicone squares (Cardiovascular Instruments Corp., PR72034-06N, 1.5 cm×1.5 cm) were pre-incubated in bovine serum (Sigma B-9433) overnight at 37°C while shaking (150 rpm) in a 12-well plastic plate. The treated silicone squares were washed with 2 ml PBS, placed in 12-well culture dishes, and 2 ml Spider medium added. *C. albicans* strains were grown overnight in YPD medium and approximately 2×10^7^ cells were added to each well. The inoculated plate was incubated at 37°C for 90 min with gentle agitation (150 rpm), either in the presence (10 µM) or absence of α pheromone, or in the presence of a 0.01% DMSO control. Squares were washed with 2 ml PBS and incubation continued in 2 ml fresh Spider medium (+/− pheromone) for 24 h or 60 h at 37°C with gentle shaking. Supernatants were removed and silicone squares allowed to dry overnight, before weighing to determine biofilm mass. In addition, biofilm dry weight was measured from biofilms grown directly on the bottom of 12-well plates, as described in Nobile *et al.* [13]. Following biofilm growth, media was replaced with PBS, biofilm cells collected by pipet, and cells filtered over a pre-weighed filter. Filters were dried overnight and weighed. Four experimental replicates were performed and significance was determined using the student's one-tailed t-test.

### Quantitation of Adherent Biofilm Cells

Pheromone-stimulated or conventional biofilms were grown on the bottom of polystyrene plates, as described above. The supernatant was removed and adherent biofilm cells obtained by scraping the well, and quantified by measuring the OD_600_.

### Quantitative Mating Assays

Opaque *MTL* **a** and *MTL*α cells expressing different selection markers were grown in SCD medium at room temperature. Approximately 2×10^7^ *MTL* **a** and *MTL*α strains were mixed together on a nitrocellulose filter on Spider medium. Cells were incubated for 48 h incubation at 25°C, then resuspended in water and plated onto selective media to quantitate mating frequency, as previously described [57].

### Quantitative PCR

*C. albicans* cells were grown as described in “Pheromone-Stimulated Biofilm Assays”. Cells were collected after pheromone treatment at room temperature for 4 h. Total RNA was isolated and cDNA synthesized as previously described [51]. Quantitative PCR was performed in a 7300 Real Time PCR System (Applied Biosystems). Signals from experimental samples were normalized to the *PAT1* gene expression level, as previously described [24].

### Northern Blot Analyses

Procedures and conditions for pre-hybridization, hybridization, washing and immunological detection of the probe with a CSPD chemofluorescent substrate for alkaline phosphatase were performed following the manufacture's recommendations (Roche Applied Science). Probes for northern blot analyses were labeled with digoxigenin-11-dUTP (Roche Applied Science) by PCR. Probes for *PBR1*, *CPH1* and *TEC1* were amplified using primers 1098/1099, 1173/1174 and 1341/1342, respectively.

### Microarray Analyses

The transcriptional profiles of white cells responding to pheromone were performed on cells grown in planktonic and biofilm culture conditions. *C. albicans* white or opaque cells were grown in Spider medium overnight at room temperature. To harvest planktonic cells, overnight cultures were added into 12 ml Lee's medium at OD_600_ = 0.3 with DMSO (control) or synthetic α pheromone (final concentration of 10 µM) and incubated at 25°C for 4 h with gently shaking (150 rpm). For biofilm cells, cells were collected from the biofilm mat after treatment with DMSO or pheromone following the method described in “Pheromone-Stimulated Biofilm Assays”.

Total RNA was isolated from cells using the Ribopure-Yeast Kit (Ambion) and treated with Turbo DNase (Ambion). cDNA was synthesized from 10 mg of total RNA using SuperScript 3 reverse transcriptase (Invitrogen) with oligos (dT) 19V and pdN9 in reaction mixtures containing 0.5 mM DTT and 0.5 mM deoxynucleoside triphosphates (aminoallyl-dUTP and deoxynucleoside triphosphates [3∶2]). RNA was hydrolyzed with 0.3 M sodium hydroxide and 0.03 M EDTA and neutralized with 0.3 M HCl to pH 7.0. cDNA was purified and recovered using a Zymo kit (Zymogen, DNA Clean & Concentrator). Samples were dried in a speed vacuum and resuspended in 9 µl of RNase-free water.

Coupling of cDNA and hybridization to microarrays was performed as previously described [48]. cDNA from cells treated with 10 µM pheromone was hybridized against cDNA from matched cells treated with a mock DMSO control. Arrays were scanned on a GenePix 4000 scanner (Axon Instruments). Profiles were quantified by using GENEPIX PRO version 3.0 and normalized using Goulphar (http://transcriptome.ens.fr/goulphar). Pairwise average linkage clustering analysis was performed using the program CLUSTER and visualized by using TREEVIEW. The *Candida* genome database (http://www.candidagenome.org/) was used to facilitate further analysis. All microarray data has been deposited into the NCBI Gene Expression Omnibus (GEO) portal under the accession number GSE44449. Gene expression changes greater than 4-fold are displayed in Figure 5. Gene expression changes greater than 2-fold or 4-fold are displayed in Figure S3. The chi squared statistical test was used to determine the significance of overlapping array data.

### Confocal Scanning Laser Microscopy

For pheromone-stimulated biofilms, 6 well plates (BD Falcon) containing 2 ml Lee's medium were inoculated with 1×10^8^ cells from overnight cultures grown in Spider medium. Biofilms were grown in static conditions at room temperature, for 24 h. Conventional biofilms were grown as in “Conventional Biofilm Assays” for 24 h. For both types of biofilm assay, α pheromone (final concentration of 10 µM) or a DMSO control was added at the beginning of the 24 h timeframe. Biofilms were stained with 50 µg/ml concanavalin A conjugated to Alexa Fluor 594 (Molecular Probes, C11253) for 1 h in the dark. Biofilms were gently washed with 1 ml PBS, then covered with water and imaged on a Nikon Eclipse C1si upright spectral imaging confocal microscope using a 40x/0.80W Nikon objective. For conA-594 visualization, excitation was at 561 nm and emission detection was at 605/60 nm. Images were acquired in a Z-stack series at 0.8 µm intervals, using Nikon EZ-C1 Version 3.80 software, and assembled into maximum intensity Z-stack projections using Nikon NIS Elements Version 3.00 software.

## Supporting Information

Figure S1 **Analysis of** ***CPH1*** **in pheromone-stimulated biofilm assays in SC5314 white cells.** Consistent within pheromone signaling in P37005 (see Figure 1) the Cph1 transcription factor is also essential for pheromone-stimulated biofilm formation in SC5314 white cells. (A) Loss of *CPH1* resulted in a significant defect in the adherence to plastic assay, and the phenotype was similar to that of the *ste2* pheromone receptor mutant (“*” represents *P*<0.001 for WT v. mutant). (B) Confocal scanning laser microscopy of biofilm formation. For each image, the top panel shows the top view and the bottom panel shows the reconstructed side view, with the plastic substrate at the bottom of the image. Scale bars are 50 µm. AB indicates addback strains in which the target gene has been reintegrated into the mutant background.
(TIF)

Figure S2 **Comparative analysis of** ***CPH1*** **and** ***TEC1*** **in conventional biofilm formation.** Conventional biofilm assays were performed in SC5314 (A–C) and P37005 (D–F) strain backgrounds both in the presence (+) and absence (−) of pheromone. Note that while *tec1* mutants are highly deficient in conventional biofilm formation, *cph1* mutants were not defective in this biofilm model. (C,F) CSLM images of biofilms formed by wildtype, *tec1*, and *cph1* mutants. Scale bars in CSLM images are 20 µm.
(TIF)

Figure S3 **Comparison of the transcriptional programs regulating conventional and pheromone-induced biofilms.** Venn diagram shows that 662 genes are induced in conventional biofilms (>2-fold, data from Nobile et al., 2012) while 486 genes are induced in pheromone-induced biofilms (>2-fold in white cells at 24 h). 128 genes are induced in both biofilm models (p = 2×10^−30^). Similarly, 187 genes are repressed in conventional biofilms (>2-fold) while 355 genes are repressed in pheromone-induced biofilms (>2-fold). 19 genes are repressed in both conditions (p = 9×10^−3^). When a 4-fold cutoff is applied, there is no overlap between the genes induced or repressed by both types of biofilm. The transcriptional changes occurring during conventional and pheromone-induced biofilms are therefore overlapping, but the genes undergoing the highest transcriptional fold changes are mostly unique to each program.
(TIF)

Figure S4 **Hgc1 is dispensable for the pheromone response and mating in opaque cells.** Cells lacking *HGC1* were tested for the ability to produce mating projections and generate mating products. (A) Deletion of the *HGC1* gene did not influence mating projection formation or mating competency. Values are the mean ± SD from two independent experiments with at least three replicates. (B) Images showing mating projections produced from *MTL* ***a*** opaque cells treated with α pheromone. Scale bar: 5 µm. (WT: CAY1477; *Δhgc1/Δhgc1*: CAY3752; *HGC1 AB*: CAY3756; *MTL*α: DSY211).
(TIF)

Table S1 **Strains used in this study.**
(DOCX)

Table S2 **Primers used in the study.**
(DOCX)

Table S3 **Microarray data.** Columns listed are in the same order as the experiments shown in Figure 5A. (P) = planktonic, (B) = biofilm, − indicates no pheromone, + indicates pheromone added. Sheet 1: All replicates and array probes, data is log2. Sheet 2: The mean value was taken of the array probes and replicates were averaged. Alternative name and description provide by the Candida Genome Database (candidagenome.org). Sheet 3: Lists of genes up- or down-regulated more than 4-fold. Sheet 4: Table of genes up- or down-regulated more than 4-fold.
(XLSX)

Table S4 **Microarray data comparing transcriptional changes in white cell biofilms (at 24 h) with those in opaque cell biofilms (at 4 h).** Genes induced and repressed under both conditions are highlighted (orange). In addition, genes involved in adhesion (yellow), biofilm formation (red), and hyphal formation (blue), are highlighted. Overall, the data shows that transcriptional changes shared between white and opaque cells are significant (p<5×10^−254^ for induced genes and p<7×10^−145^ for repressed genes).
(XLSX)
