# Supplementary figures and images for: Genetic Control of Conventional and Pheromone-Stimulated Biofilm Formation in Candida albicans
Source: PLoS Pathog. 2013 Apr 18;9(4):e1003305. doi: 10.1371/journal.ppat.1003305 (PMC3630098; doi:10.1371/journal.ppat.1003305)

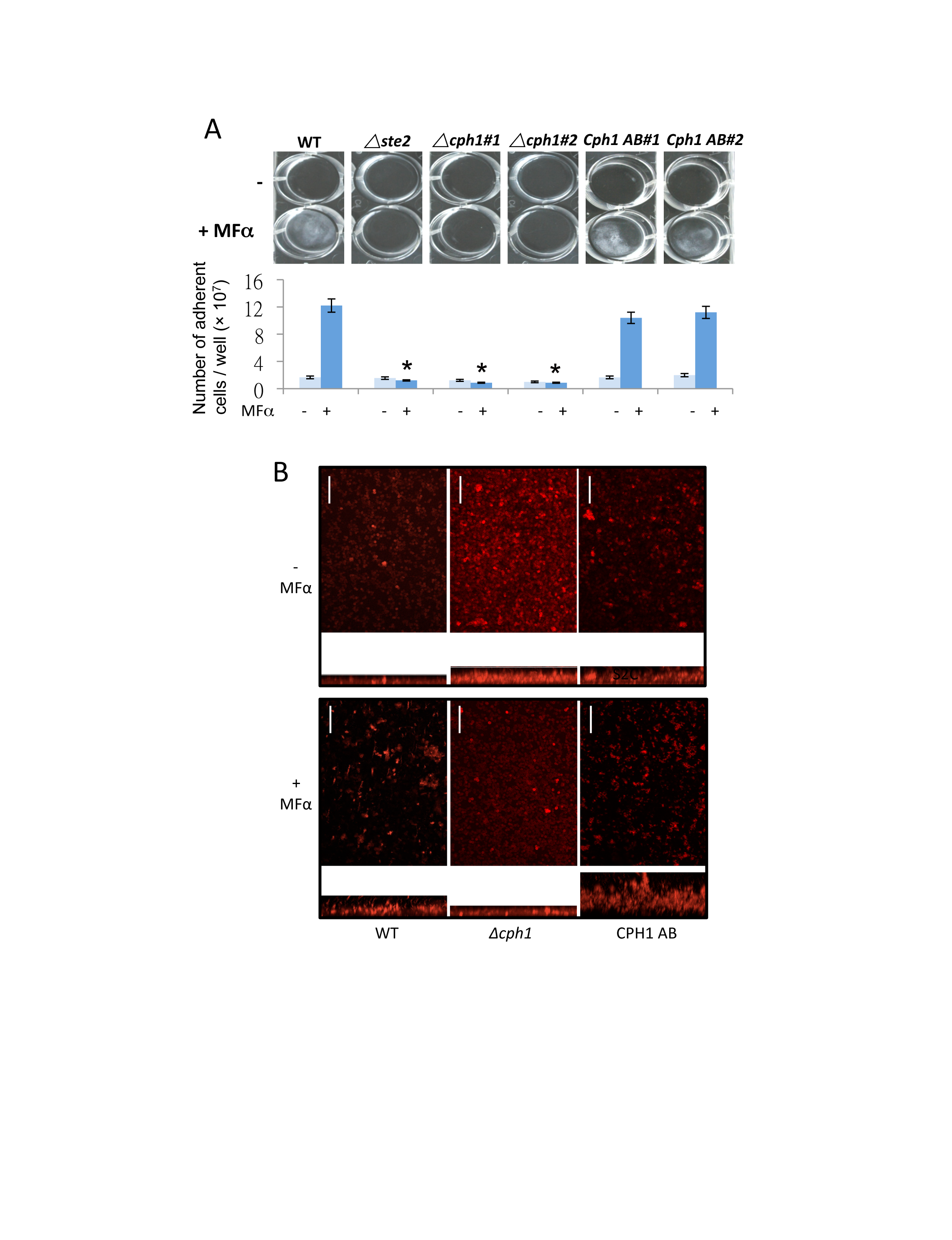

Supplement: Figure S1 — Analysis of CPH1 in pheromone-stimulated biofilm assays in SC5314 white cells. Consistent within pheromone signaling in P37005 (see Figure 1) the Cph1 transcription factor is also essential for pheromone-stimulated biofilm formation in SC5314 white cells. (A) Loss of CPH1 resulted in a significant defect in the adherence to plastic assay, and the phenotype was similar to that of the ste2 pheromone receptor mutant (“*” represents P<0.001 for WT v. mutant). (B) Confocal scanning laser microscopy of biofilm formation. For each image, the top panel shows the top view and the bottom panel shows the reconstructed side view, with the plastic substrate at the bottom of the image. Scale bars are 50 µm. AB indicates addback strains in which the target gene has been reintegrated into the mutant background. (TIF) [file ppat.1003305.s001.tif]

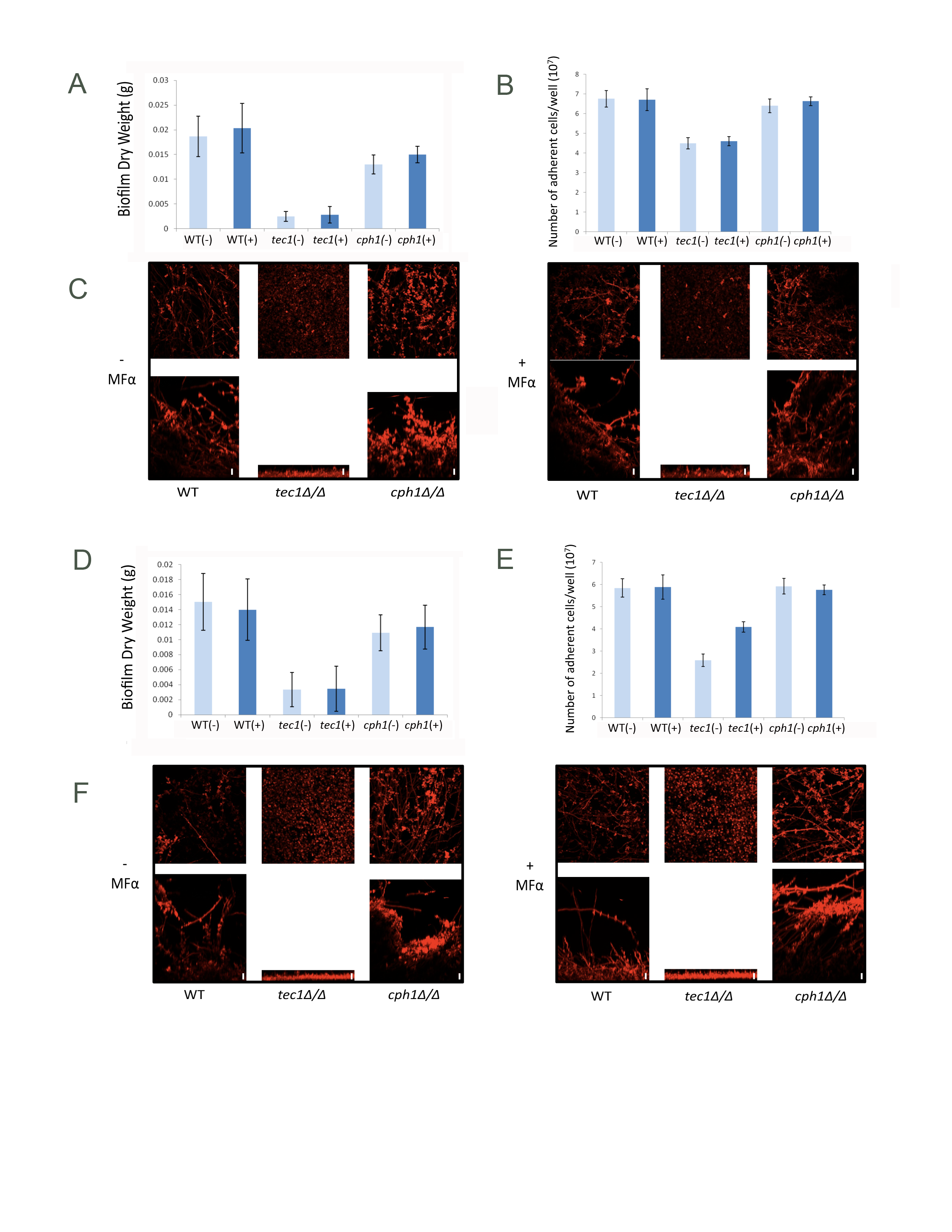

Supplement: Figure S2 — Comparative analysis of CPH1 and TEC1 in conventional biofilm formation. Conventional biofilm assays were performed in SC5314 (A–C) and P37005 (D–F) strain backgrounds both in the presence (+) and absence (−) of pheromone. Note that while tec1 mutants are highly deficient in conventional biofilm formation, cph1 mutants were not defective in this biofilm model. (C,F) CSLM images of biofilms formed by wildtype, tec1, and cph1 mutants. Scale bars in CSLM images are 20 µm. (TIF) [file ppat.1003305.s002.tif]

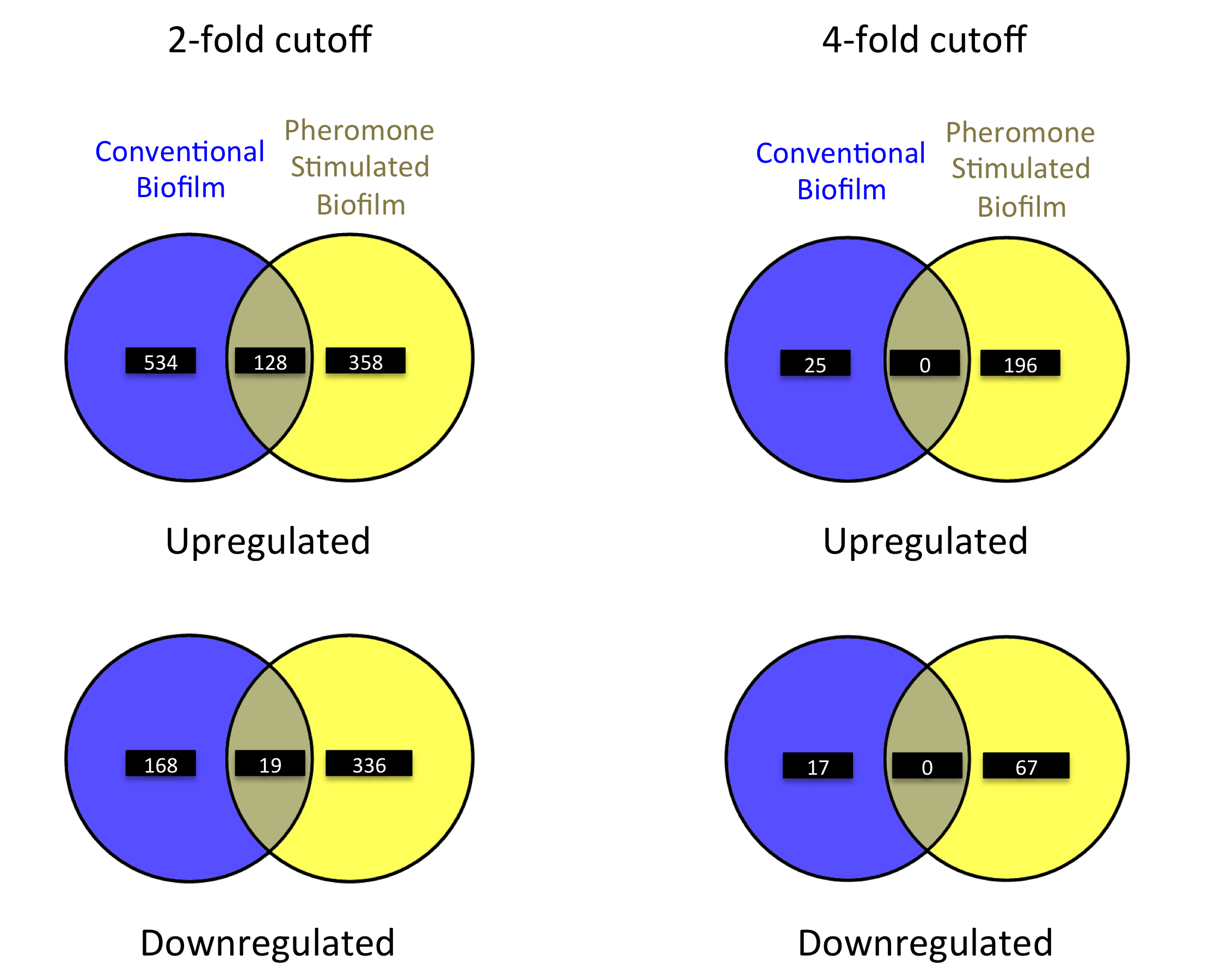

Supplement: Figure S3 — Comparison of the transcriptional programs regulating conventional and pheromone-induced biofilms. Venn diagram shows that 662 genes are induced in conventional biofilms (>2-fold, data from Nobile et al., 2012) while 486 genes are induced in pheromone-induced biofilms (>2-fold in white cells at 24 h). 128 genes are induced in both biofilm models (p = 2×10^−30^). Similarly, 187 genes are repressed in conventional biofilms (>2-fold) while 355 genes are repressed in pheromone-induced biofilms (>2-fold). 19 genes are repressed in both conditions (p = 9×10^−3^). When a 4-fold cutoff is applied, there is no overlap between the genes induced or repressed by both types of biofilm. The transcriptional changes occurring during conventional and pheromone-induced biofilms are therefore overlapping, but the genes undergoing the highest transcriptional fold changes are mostly unique to each program. (TIF) [file ppat.1003305.s003.tif]

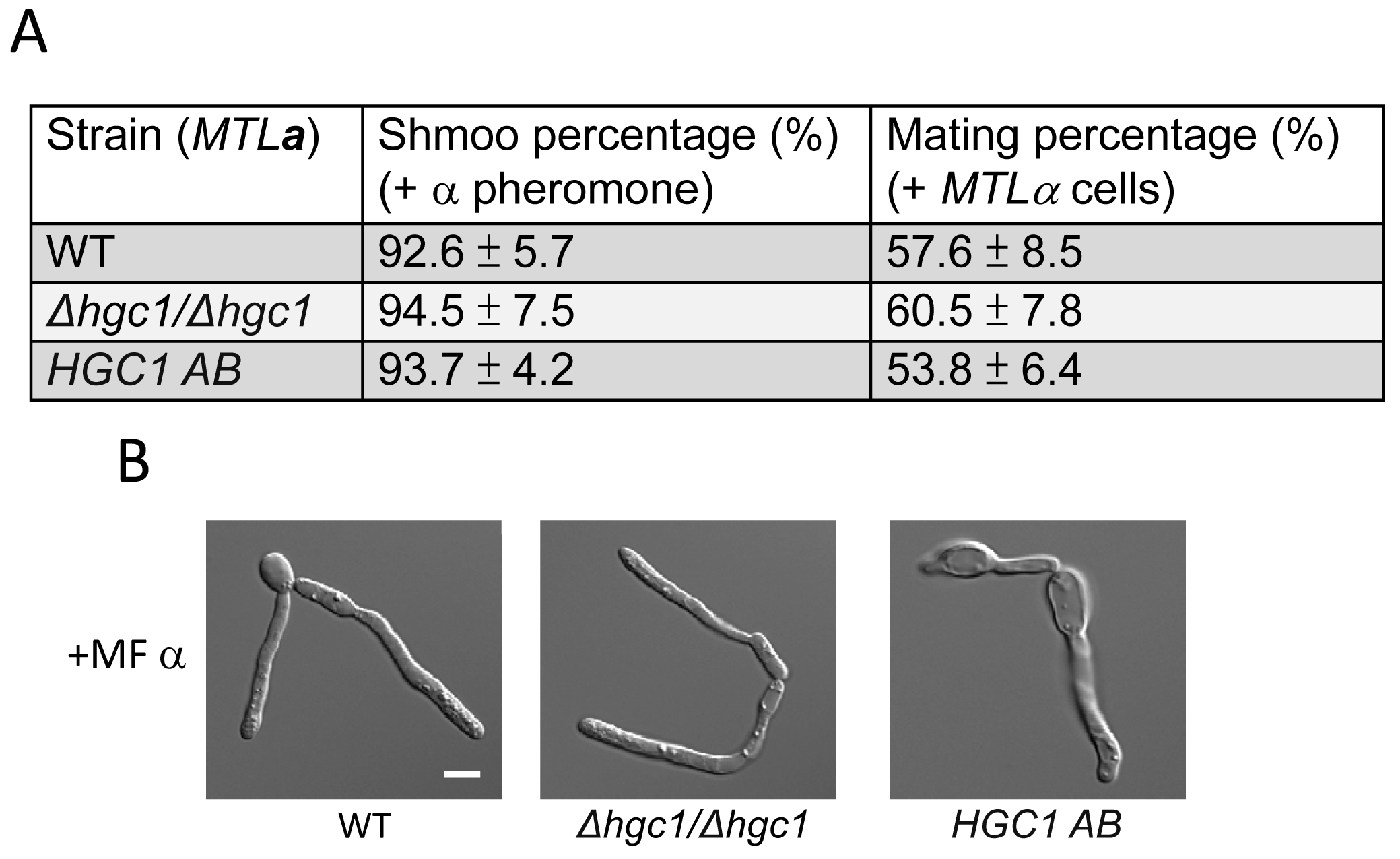

Supplement: Figure S4 — Hgc1 is dispensable for the pheromone response and mating in opaque cells. Cells lacking HGC1 were tested for the ability to produce mating projections and generate mating products. (A) Deletion of the HGC1 gene did not influence mating projection formation or mating competency. Values are the mean ± SD from two independent experiments with at least three replicates. (B) Images showing mating projections produced from MTL a opaque cells treated with α pheromone. Scale bar: 5 µm. (WT: CAY1477; Δhgc1/Δhgc1: CAY3752; HGC1 AB: CAY3756; MTLα: DSY211). (TIF) [file ppat.1003305.s004.tif]
