# Supplementary material for: Genetic Control of Conventional and Pheromone-Stimulated Biofilm Formation in Candida albicans
Source: PLoS Pathog. 2013 Apr 18;9(4):e1003305. doi: 10.1371/journal.ppat.1003305 (PMC3630098; doi:10.1371/journal.ppat.1003305)
Supplement: Table S1 — Strains used in this study. (DOCX) [file ppat.1003305.s005.docx]

**Table S1**: Strains used in this study. Only one representative isolate listed here.

* Indicates that isolates are P37005 strain background. All other strains are SC5314-derived.

| Strains | Genotype | White/ opaque | Source |
| --- | --- | --- | --- |
| CAY716* | **a**/**a** (wildtype P37005) | White | [23] |
| CAY1234* | **a/a** *ste2/ste2* | White | [51] |
| CAY1238 | **a/a** *wor1/wor1* | White | This study |
| CAY1477* | **a/a (**opaque P37005) | Opaque | [51] |
| CAY1478 | **a/a** *ste2/ste2* | Opaque | [51] |
| CAY2504 | **a**/**a** *tec1/tec1::SAT* | White | This study |
| CAY2506* | **a**/**a** *tec1/tec1::SAT* | White | This study |
| CAY2687 | **a**/**a** *tec1/tec1* | Opaque | This study |
| CAY2689* | **a**/**a** *tec1/tec1* | Opaque | This study |
| CAY2748 | **a**/**a** *tec1/tec1::TEC1* | White | This study |
| CAY2750* | **a**/**a** *tec1/tec1::TEC1* | White | This study |
| CAY2773 | **a**/**a** *tec1/tec1::TEC1* | Opaque | This study |
| CAY2775* | **a**/**a** *tec1/tec1::TEC1* | Opaque | This study |
| CAY2895 | **a**/**a** *cph1/cph1* | White | This study |
| CAY2899* | **a**/**a** *cph1/cph1* | White | This study |
| CAY2943 | **a**/**a** *cph1/cph1* | Opaque | This study |
| CAY2947* | **a/a** *cph1/cph1* | Opaque | This study |
| CAY3025 | **a**/**a** *cph1/cph1::CPH1* | White | This study |
| CAY3028* | **a**/**a** *cph1/cph1::CPH1* | White | This study |
| CAY3043 | **a**/**a** *cph1/cph1::CPH1* | Opaque | This study |
| CAY3046* | **a**/**a** *cph1/cph1::CPH1* | Opaque | This study |
| CAY3771 | **a/a** *leu2/leu2 WH11/WH11-mCherry* | White | This study |
| CAY3445* | **a/a** *orf19.7167/orf19.7167::SAT1* | White | This study |
| CAY3447* | **a/a** *orf19.7170/orf19.7170::SAT1* | White | This study |
| CAY3465* | **a/a** *hgc1/hgc1::SAT1* | White | This study |
| CAY3488* | **a/a** *hgc1/hgc1* | White | This study |
| CAY3526 | **a/a** *efg1/efg1* | White | This study |
| CAY3583 | **a/a** *arg::hisG/arg::hisG brg1::HIS1/brg1::LEU:2* | White | This study |
| CAY3593 | **a/a** *ndt80/ndt80::Ndt80::SAT1* | White | This study |
| CAY3670 | **a/a** *arg1::hisG/arg1::hisG rob1::HIS1/rob1::LEU2* | White | This study |
| CAY3672 | **a/a** *arg1::hisG/arg1::hisG bcr1::HIS1/bcr1::LEU2* | White | This study |
| CAY3687* | **a/a** *cfl11/cfl11::SAT1* | White | This study |
| CAY3689* | **a/a** *pbr1/pbr1::SAT1* | White | This study |
| CAY3693* | **a/a** *orf19.7305/orf19.7305::SAT1* | White | This study |
| CAY3702* | **a/a** *hgc1/hgc1::HGC1* | White | This study |
| CAY3752* | **a/a** *hgc1/hgc1* | Opaque | This study |
| CAY3756* | **a/a** *hgc1/hgc1::HGC1* | Opaque | This study |
| CAY3802 | **a/a** *arg::hisG/arg::hisG brg1::HIS1/BRG1::SAT1* | White | This study |
| CAY3805 | **a/a** *arg1::hisG/arg1::hisG rob1::HIS1/ROB1::SAT1* | White | This study |
| CAY3816 | **a/a** *arg4/arg4 ndt80::HIS1/ndt80::HIS1 RP10/rp10::pCIpACT1-SUN41* | White | This study |
| CAY3818 | **a/a** *arg4/arg4 ndt80::HIS1/ndt80::HIS1 RP10/rp10::pCIpACT1-CHT3* | White | This study |
| CAY3858 | **a/a** *arg1::hisG/arg1::hisG bcr1::HIS1/BCR1::SAT1* | White | This study |
| CAY4026 | **a/a** *efg1/efg1::EFG1::HYG* | White | This study |
| DSY211 | **α/α** *leu2::hisG/leu2::hisG his1::hisG/his1::hisG::SAT1* | Opaque | [57] |
| RBY520 | **a/a** *ndt80/ndt80* | White | This study |
| RBY717 | **a**/**a** *ura3::imm434/URA iro1::imm434/IRO1* | White | [6] |
| RBY731 | **a/a** *ura3::imm434/URA iro1::imm434/IRO1* | Opaque | [6] |
| RBY1107 | **a/a** *ste2::LEU2/ste2::HIS* | White | [57] |
| RBY1132 | **a/a** *arg1/arg1 his1/his1 leu2/leu2* | White | [13] |
| TF22 | **a/α** *arg1::hisG/arg1::hisG brg1::HIS1/brg1::LEU2* | White | [13] |
| TF95 | **a/α** *arg1::hisG/arg1::hisG ndt80::HIS1/ndt80::LEU2* | White | [13] |
| TF110 | **a/α** *arg1::hisG/arg1::hisG rob1::HIS1/rob1::LEU2* | White | [13] |
| TF115 | **a/α** *arg1::hisG/arg1::hisG tec1::HIS1/tec1::LEU2* | White | [13] |
| TF137 | **a/α** *arg1::hisG/arg1::hisG bcr1::HIS1/bcr1::LEU2* | White | [13] |
| TF156 | **a/α** *arg1::hisG/arg1::hisG rob1::HIS1/rob1::LEU2* | White | [13] |
| OHY13 | **a/α** *arg1::hisG/arg1::hisG* | White | [13] |
| TF22a | **a/***Δ*α *arg1::hisG/arg1::hisG brg1::HIS1/brg1::LEU2* | White | This study |
| TF95a | **a/***Δ*α *arg1::hisG/arg1::hisG ndt80::HIS1/ndt80::LEU2* | White | This study |
| TF110a | **a/***Δ*α *arg1::hisG/arg1::hisG rob1::HIS1/rob1::LEU2* | White | This study |
| TF115a | **a/***Δ*α *arg1::hisG/arg1::hisG tec1::HIS1/tec1::LEU2* | White | This study |
| TF137a | **a/***Δ*α *arg1::hisG/arg1::hisG bcr1::HIS1/bcr1::LEU2* | White | This study |
| TF156a | **a/***Δ*α *arg1::hisG/arg1::hisG rob1::HIS1/rob1::LEU2* | White | This study |
| OHY13a | **a/***Δ*α *arg1::hisG/arg1::hisG* | White | This study |
