# Supplementary material for: Genetic Control of Conventional and Pheromone-Stimulated Biofilm Formation in Candida albicans
Source: PLoS Pathog. 2013 Apr 18;9(4):e1003305. doi: 10.1371/journal.ppat.1003305 (PMC3630098; doi:10.1371/journal.ppat.1003305)
Supplement: Table S2 — Primers used in the study. (DOCX) [file ppat.1003305.s006.docx]

**Table S2**: Oligonucleotides used in this study.

| Name | Sequence (5’-3’) |
| --- | --- |
| 1098 | tgctgcttttttcagtttatttttcc |
| 1099 | cctggagtaacgtaatcaccaatg |
| 1167 | ggcgccgggcccgaattagacagggaccttgac |
| 1168 | ggcgcgctcgagggagtagcttgcgacatcat |
| 1169 | ggagcgccgcggcttctatgaataccggatatgg |
| 1170 | ggccgcgagctccatcgccagcatcttcaatattg |
| 1173 | gatccagtgtcttctgttgg |
| 1174 | atcacttaaaccagggaacc |
| 1187 | ggagcggggcccgctggttgttcggtatttcaaatc |
| 1188 | ggagcgctcgagcatcaatccgtgatcatgttgac |
| 1189 | ggagcggagctcatccataacatgcctgtcgtagg |
| 1194 | ggagcggggcccgggttaggtttcagcaacgaatg |
| 1195 | ggagcgctcgaggattgggtgctattgtatcaatggtat |
| 1196 | ggagcgccgcggggtactctgttcttcgttatttcac |
| 1197 | ggagcggagctccccagaaagcaccgcaaata |
| 1218 | ggagcggggcccgtggtccattccaacttctaactca |
| 1219 | ggagcgctcgagatttcaaggaaggaatggaccc |
| 1220 | ggagcgccgcggttgggcgcaacgttacattt |
| 1221 | ggagcggagctcgggagaattgattagttgcatatttg |
| 1226 | ggagcggggccctgtggtggtcgtttcatcattt |
| 1202 | ggagcggggcccgggctttagaggcaacttgttg |
| 1203 | ggagcgctcgagcctaagagatgaagggtgggtt |
| 1204 | ggagcgccgcggccactcactcatctcccttccc |
| 1205 | ggagcggagctcccttcgtgaagatggaggttacac |
| 1210 | ggagcggggcccgagagaaattctgtcctcctccc |
| 1211 | ggagcgctcgagagttagctgtcgttgttgttgttg |
| 1212 | ggagcgccgcggggagaatggagaaagatgttgttatt |
| 1213 | ggagcggagctcgagaccgaagtaacaattcaggac |
| 1227 | ggagcgctcgagcccatttatacccatgctaggg |
| 1228 | ggagcgccgcgggcttactgtttgatgttgttgatcc |
| 1229 | ggagcggagctctcgttgctgaaacctaacccc |
| 1334 | ggagcggggcccgggtgaacgcatggttataatgt |
| 1335 | ggagcgctcgagcttcatcatcatttacaatcgaacc |
| 1336 | ggagcgggtacccccgttacccgaaacacacata |
| 1337 | ggagcggggcccggcgaaagagtgtattaggcag |
| 1338 | ggagcggagctcagcttagatggggttgccttac |
| 1341 | gccacaccaagtcaatacaaag |
| 1342 | aggtataggaaacccagaagcgtc |
| 1346 | ggagcgccgcgggatgaatagatacagagagaaagagaaag |
| 1419 | ggagcggcggccgctcttcaattctcgatttcctcgtg |
| 1420 | ggagcggagctcagacgacgacgatggaagaaa |
| 1845 | ggcgccctcgaggttcaaaacttcatctcaaacctc |
| 1838 | cggccgggtaccattgccctacccatctactcgc |
| 1839 | ggccgcgggccctgtcaatggatttgggagaag |
| 1875 | gccggcgggccccattctttgggctaattgtttc |
| 1876 | ggcgccgtcgacgatgataatttgaaactccagg |
| 1878 | gccggcgggccccatcatcctgtcaattcaaag |
| 1879 | ggcgccctcgagcccaactagcaatagaaatag |
| 1881 | gccggcgggccccctttctttcccttgtatctg |
| 1882 | ggcgccctcgagcctacatgaacaacgaagatg |
| 2115 | ggagcgccgcgggtaagtggagggttttcgtacgtg |
| MBL 660 | gtcagcggccgcatccctgccactggaaataagtggtggtagtag |
| MBL 661 | gttacacccgggcattggaattattgtctaacctgcc |
| MBL 662 | cacggcgcgcctagcagcgggtacatacaaaactggttattgtagcagg |
| MBL 663 | ctcggacccgggccttgggtagtaaaggtaaagcc |
| UP2 | ccgctgctaggcgcgccgtgaccagtgtgatggatatctgc |
| UP5 | gcagggatgcggccgctgacagctcggatccactagtaacg |
